# RNA m^5^C modification upregulates E2F1 expression in a manner dependent on YBX1 phase separation and promotes tumor progression in ovarian cancer

**DOI:** 10.1038/s12276-024-01184-4

**Published:** 2024-03-01

**Authors:** Xiaoyi Liu, Qinglv Wei, Chenyue Yang, Hongyan Zhao, Jie Xu, Youchaou Mobet, Qingya Luo, Dan Yang, Xinzhao Zuo, Ningxuan Chen, Yu Yang, Li Li, Wei Wang, Jianhua Yu, Jing Xu, Tao Liu, Ping Yi

**Affiliations:** 1 Department of Obstetrics and Gynecology, The Third Affiliated Hospital of Chongqing Medical University, Chongqing, 401120 China; 2 Chongqing Key Laboratory of Child Infection and Immunity, Children’s Hospital of Chongqing Medical University, Chongqing, 400014 China; 3 Department of Pathology, Southwest Hospital, Army Medical University, Chongqing, 400038 China; 4 Department of Obstetrics and Gynecology, Daping Hospital, Army Medical University, Chongqing, 400042 China; 5 Institute of Life Sciences, Chongqing Medical University, Chongqing, 400016 China; 6 Department of Hematology and Hematopoietic Cell Transplantation, City of Hope National Medical Center, Duarte, CA 91010 USA

**Keywords:** Gynaecological cancer, Oncogenes

## Abstract

5-Methylcytosine (m^5^C) is a common RNA modification that modulates gene expression at the posttranscriptional level, but the crosstalk between m^5^C RNA modification and biomolecule condensation, as well as transcription factor-mediated transcriptional regulation, in ovarian cancer, is poorly understood. In this study, we revealed that the RNA methyltransferase NSUN2 facilitates mRNA m^5^C modification and forms a positive feedback regulatory loop with the transcription factor E2F1 in ovarian cancer. Specifically, NSUN2 promotes m^5^C modification of E2F1 mRNA and increases its stability, and E2F1 binds to the *NSUN2* promoter, subsequently reciprocally activating *NSUN2* transcription. The RNA binding protein YBX1 functions as the m^5^C reader and is involved in NSUN2-mediated E2F1 regulation. m^5^C modification promotes YBX1 phase separation, which upregulates E2F1 expression. In ovarian cancer, NSUN2 and YBX1 are amplified and upregulated, and higher expression of NSUN2 and YBX1 predicts a worse prognosis for ovarian cancer patients. Moreover, E2F1 transcriptionally regulates the expression of the oncogenes MYBL2 and RAD54L, driving ovarian cancer progression. Thus, our study delineates a NSUN2-E2F1-NSUN2 loop regulated by m^5^C modification in a manner dependent on YBX1 phase separation, and this previously unidentified pathway could be a promising target for ovarian cancer treatment.

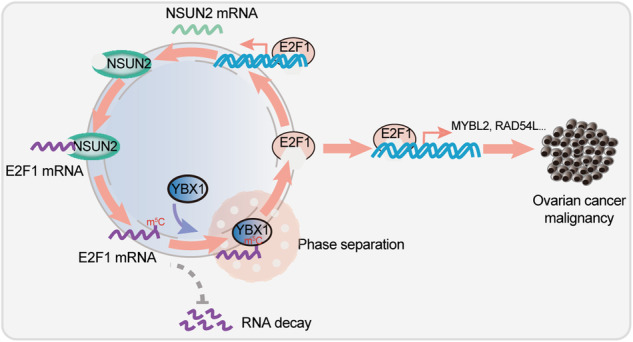

## m5C modification: a new pathway in ovarian cancer treatment

Ovarian cancer is the most lethal women’s reproductive system cancer globally, largely due to the absence of early detection techniques. Scientists have discovered that a gene named NSUN2, often found in excess in ovarian cancer, is vital for the cancer’s growth. The research, led by P.Y. and T.L., revealed that NSUN2 encourages the expansion and spread of ovarian cancer cells. They also found that NSUN2 controls the activity of another gene, E2F1, through a method called m5C modification (a process that alters gene expression). This method is essential for E2F1’s RNA stability, a significant factor in cancer growth. The study indicates that focusing on NSUN2 and E2F1 could be a potential treatment approach for ovarian cancer. This summary was initially drafted using artificial intelligence, then revised and fact-checked by the author.

## Introduction

Ovarian cancer has the highest mortality rate among gynecological cancers worldwide, and epithelial ovarian cancer is the most common type of ovarian cancer^1,2^. The lack of reliable early screening tests for ovarian cancer delays its diagnosis, and approximately 50% of patients die within five years after diagnosis^2–4^. Despite recent therapeutic advances, ovarian cancer still has poor outcomes due to the unclear gene regulatory network underlying its pathogenesis.

Recent studies have shown close associations of RNA modifications with the initiation and progression of diseases, including cancer^5–9^. As an epigenetic regulator of RNA metabolism, RNA modifications play an important role in the posttranscriptional modulation of gene expression^10^. *N*^6^-methyladenosine (m^6^A) is the most abundant RNA modification and has been implicated in various cancers. In ovarian cancer, the expression of multiple m^6^A regulators can be used to predict an adverse prognosis, and these molecules regulate the metabolism of key oncogenes or tumor suppressors^5,11–14^. 5-Methylcytosine (m^5^C) is another dynamic and reversible RNA methylation modification occurring extensively on ribosomal RNAs (rRNAs), transfer RNAs (tRNAs) and message RNAs (mRNAs)^15–17^. The deposition of m^5^C on RNA is catalyzed by a set of proteins, including the methyltransferases NOP2/Sun RNA methyltransferase family members 1–7 (NSUN1–7) and DNA methyltransferase 2 (DNMT2, also named TRDMT1)^18^. Among these methyltransferases, NSUN2 and NSUN6 primarily direct the m^5^C modification of mRNAs^18–20^. m^5^C modification affects numerous processes of RNA metabolism, such as nuclear export, decay and translation^21–25^. The RNA-binding proteins Aly/REF export factor (ALYREF) and Y-box binding protein 1 (YBX1) are two m^5^C readers that orchestrate RNA nuclear export and RNA stability, respectively^21,22^. Several studies revealed that RNA m^5^C modification is involved in tumorigenesis and metastasis in cancers^22,23,26^. In bladder cancer, RNA m^5^C modification stabilizes pyruvate kinase muscle isozyme M2 (PKM2) mRNA concomitant with increased glycolytic activity^27^. NSUN2-induced m^5^C modification of the mRNA of the oncogene heparin-binding growth factor (HDGF) also participates in the pathogenesis of bladder cancer^22^. In hepatocellular carcinoma, the long noncoding RNA H19 is subject to m^5^C modification, which contributes to poor tumor differentiation^28^. However, the role of RNA m^5^C modification in ovarian cancer and its interaction with transcription factors remain elusive.

In this study, we revealed the crucial functions of NSUN2-mediated m^5^C modification of mRNAs in ovarian cancer. NSUN2 is aberrantly upregulated in ovarian cancer and promotes the malignancy of ovarian cancer through regulating the expression of the oncogenic driver E2F transcription factor 1 (E2F1) in an m^5^C-dependent manner, and E2F1 facilitates the transcription of NSUN2 as well as MYBL2 and RAD54L. Notably, YBX1 recognizes m^5^C-modified E2F1 mRNA and increases its stability. We also revealed that m^5^C modification promotes the phase separation of YBX1, which is indispensable for E2F1 upregulation. Thus, our findings highlight the effect of mRNA m^5^C modification and the YBX1 condensation-dependent regulatory mechanism of the NSUN2-E2F1-NSUN2 loop in ovarian cancer.

## Materials and methods

### Cell culture

HEK293T, A2780, SKOV3, and OVCAR3 cells were purchased from the American Type Culture Collection (Manassas, VA, USA). HEK293T and SKOV3 cells were cultured in DMEM containing 10% fetal bovine serum and 1% penicillin/streptomycin, while A2780 and OVCAR3 cells were cultured in RPMI 1640 medium containing 10% fetal bovine serum and 1% penicillin/streptomycin (Thermo Fisher Scientific). All cells were cultured in a cell incubator at 37 °C and 5% CO_2_.

### Tumor samples

Samples of serous epithelial ovarian cancer tissues, fallopian tube epithelial tissues and normal ovarian epithelial tissues were obtained from the Third Affiliated Hospital of Chongqing Medical University and Daping Hospital between 2016 and 2022. All of these samples were confirmed by pathological examination. All experiments involving human samples were approved by the Accreditation Committee of the Third Affiliated Hospital of Chongqing Medical University and the Institutional Review Board of DAPING Hospital at Army Medical University.

### Plasmids, transfection and lentiviral transduction

Complementary sense and antisense oligonucleotides encoding shRNAs targeting NSUN2, YBX1 and E2F1 were synthesized, annealed, cloned and inserted into the pLKO.1 vector. The sequences of the oligonucleotides are shown in Supplementary Table 1. The NSUN2 overexpression plasmid was constructed by inserting the full-length NSUN2 coding sequence into the pCDH vector. The FLAG-tagged human NSUN2 plasmid (pEnter-NSUN2-FLAG-His) was purchased from Vigene Biosciences, Inc. The FLAG-tagged human E2F1 plasmid (pcDNA3.1-E2F1-FLAG) was purchased from Miaoling Biosciences, Inc. The NSUN2-MUT expression plasmid was generated by introducing point mutations into the pEnter-NSUN2-FLAG-His plasmid with the Mut Express MultiS Fast Mutagenesis Kit V2.

Plasmid transfection was performed using Lipofectamine 2000 (Thermo Fisher Scientific). For lentivirus production, lentiviral vectors were cotransfected into HEK293T cells with the packaging plasmid psPAX2 and the envelope plasmid pMD2.G using Lipofectamine LTX (Thermo Fisher Scientific). Infectious lentiviral particles were harvested 48 h after transfection, filtered through 0.45 μm PVDF filters, and used to transduce ovarian cancer cells.

### Cloning, expression and purification of proteins

The tGFP-YBX1 (1–324) fusion sequence with an N-terminal His-tag and an N-terminal MBP tag upstream of a SARS Mpro protease cleavage site was cloned and inserted into the pMAT9s vector. The constructs were subsequently transformed into *E. coli* BL21 cells for expression of the recombinant protein. After 18 h of induction with 0.5 mM IPTG at 16 °C, 2 L of the *E. coli* culture was harvested, and bacterial cells were collected by centrifugation for 15 min at 4000 rpm. The *E. coli* cell pellet was resuspended in 35 ml of lysis buffer (50 mM Tris-HCl (pH 8.5), 2 M KCl, 5% glycerol, 10 mM MgCl_2_, 50 µl of Benzonase nuclease, 1 mM DTT, and 1 mM PMSF). The cell pellet was lysed by high-pressure homogenization followed by centrifugation for 60 min at 20,000 rpm and 4 °C. The supernatant was then loaded onto dextrin sepharose (MBP Trap HP, GE Healthcare), and the bound proteins were eluted with elution buffer (50 mM Tris-HCl (pH 8.5), 2 M KCl, 5% glycerol, 10 mM MgCl_2_, 50 mM maltose) and concentrated into a 500 µl volume using an Amicon Ultra Centrifugal Filter Unit. Benzonase nuclease (50 µl) was added to digest the nucleic acids at 4 °C overnight. The target protein was subsequently purified with heparin sepharose (HiTrap Heparin HP, GE Healthcare) and size exclusion chromatography (Superdex 200, GE Healthcare). The purified protein was stored in storage buffer (50 mM Tris-HCl (pH 7.4), 500 mM KCl, 5% glycerol, 1 mM DTT) at −80 °C.

### In vitro phase separation assays

For droplet formation, proteins were diluted from a stock solution into a buffer containing a final concentration of 5% PEG 4000, 25 mM Tris-HCl (pH 7.4), and 150 mM KCl at 4 °C. All the components were mixed in a microtube to induce uniform phase separation. To observe the propensity of RNA to partition into condensates, we resuspended RNA in RNase-free water at the indicated concentrations. The samples were added to glass bottom cell culture dishes (Biosharp, BS-15-GJM). Images were acquired using an A1R HD25 confocal microscope (Nikon) with a 60× oil immersion objective.

### Fluorescence recovery after photobleaching (FRAP) assay

In vitro droplets were formed by diluting the stock protein solution to a final concentration of 10 µM in 25 mM Tris-HCl (pH 7.4) buffer containing 150 mM KCl. The FRAP assay was performed using an A1R+ confocal microscope (Nikon) with a 60× oil immersion objective. For the FRAP assay of YBX1 droplets, spots with a diameter of 1 mm in 2 mm droplets were photobleached with 70% laser power using a 488 nm laser. Time-lapse images were acquired after photobleaching. The images were analyzed with Nikon software, and FRAP curves were generated and plotted using Prism software.

### RNA isolation and RT‒qPCR

Total RNA was extracted from cells using TRIzol reagent following the manufacturer’s instructions. Equal amounts of RNA were reverse transcribed to cDNA with HiScript II Q RT SuperMix (Vazyme). RT‒qPCR was performed with a Bio-Rad CFX96 real-time PCR system using the indicated primers and SYBR qPCR Master Mix (Vazyme). The sequences of the primers used are shown in Supplementary Table 1.

### Western blot analysis

Proteins were extracted using lysis buffer, separated via SDS‒PAGE, and transferred onto PVDF membranes; the membranes were then blocked with 5% nonfat milk in TBS/Tween 20 and then incubated with the indicated primary and secondary antibodies. The antibodies used were as follows: anti-NSUN2 (1:500 dilution; Proteintech), anti-YBX1 (1:2000 dilution; Proteintech), anti-E2F1 (1:1000 dilution; Proteintech), anti-ALYREF (1:500 dilution; Proteintech), anti-Lin28B (1:500 dilution; Proteintech), anti-GAPDH (1:20000 dilution; Proteintech), anti-Tubulin (1:1000 dilution; Proteintech) and anti-Flag (1:2000 dilution; MBL).

### Cell growth assays

For the cell viability assays, cells were seeded in 96-well plates at 2000 cells per well and cultured for 0, 24, 48, 72, and 96 h. After the cells were incubated with CCK-8 (Dojindo) for 2 h, the absorbance of each well at 450 and 630 nm was measured by a microplate reader. For the colony formation assays, cells were seeded in 6-well plates at 3000 cells per well, and the medium was refreshed every 3 days. After culture for 2 weeks, the colonies were fixed with methanol and stained with crystal violet.

### Transwell assays

First, 10^5^ cells in 400 μl of serum-free culture medium were seeded into Transwell inserts (Corning), and the inserts were then inserted in a 24-well plate. Then, 600 μl of culture medium supplemented with 20% FBS was added to each lower chamber. After culture for 24 h, the migrated cells were fixed with methanol and stained with crystal violet. For the invasion assays, 50 μl of serum-free medium containing 10% Matrigel was added to the upper chamber before the cells were plated. Migrated or invaded cells were imaged and counted under a microscope with a 20× objective.

### Animal models

All animal studies were approved by the Institutional Animal Care and Use Committee of Chongqing Medical University and were performed according to the institutional ethical guidelines for animal experiments. For the tumorigenesis assay, 10^6^ OVCAR3 cells transduced with shRNAs or the corresponding empty vectors were suspended in 100 μl of PBS and injected into the right axillae of nude mice at the age of 6 weeks. Tumor growth was measured and tumor volumes were calculated every 3 days beginning on the 7th day after cell inoculation. The mice were sacrificed after 3 weeks, after which the xenografts were weighed. For the metastasis assays, 5 × 10^5^ OVCAR3 cells transduced with shRNAs or the corresponding empty vectors were suspended in 300 μl of PBS and injected intraperitoneally into nude mice. The mice were sacrificed after 3 weeks, and the metastatic nodules were counted.

### Dot blot assay

RNA was mixed in denaturation buffer at 65 °C for 5 min for denaturation and immediately cooled on ice. An equal volume of precooled 20× SSC was added to the RNA sample and mixed well. Then, the RNA was spotted onto an Amersham Hybond-N+ membrane (GE Healthcare) using a Bio-Dot apparatus (Bio-Rad) and crosslinked by UV light. The membrane was blocked with 5% nonfat milk in PBST for 1 h at room temperature and incubated with an anti-m^5^C primary antibody (Abcam) overnight at 4 °C. After washing with PBST, the membrane was incubated with an HRP-conjugated secondary antibody for 1 h and developed using a chemiluminescence image analysis system. For the loading control, methylene blue was used to stain the same RNA samples.

### Immunofluorescence (IF) and RNA fluorescence in situ hybridization (RNA-FISH)

For IF, cells were fixed with 4% paraformaldehyde. Fixed cells were permeabilized with 0.2% Triton X-100 in PBS and blocked with 1% BSA in PBST. Then, the cells were incubated with an anti-NSUN2 antibody (Proteintech) overnight at 4 °C. After washing with PBS, the cells were incubated with the secondary antibody (1:200, goat anti-rabbit IgG (H + L) Alexa Fluor 488, Abcam) for 1 h. DAPI was added to the cells, and images were acquired with a fluorescence microscope. For RNA-FISH combined with IF staining, cells were fixed with 4% paraformaldehyde. An IF-FISH Kit (BersinBio) was subsequently used for RNA in situ hybridization and immunofluorescence staining according to the manufacturer’s instructions. The antibodies used were as follows: anti-YBX1 (1:100 dilution; Proteintech) and Alexa Fluor 488-conjugated goat anti-rabbit IgG (H + L) (1:200; Abcam).

### RNA sequencing (RNA-seq)

Total RNA was isolated using TRIzol reagent, and poly(A) RNA was subsequently purified by using the PolyATtract mRNA Isolation System and used to generate cDNA libraries according to the instructions of the NEBNext Ultra RNA Library Prep Kit for Illumina (New England Biolabs). All the samples were sequenced on an Illumina NovaSeq platform.

### RNA immunoprecipitation and high-throughput sequencing (RIP-seq)

Cells were collected and washed with PBS, after which IP lysis buffer (150 mM KCl, 25 mM Tris (pH 7.4), 5 mM EDTA, 0.5 mm DTT, 0.5% NP-40, 1× protease inhibitor, and 1 U/μl RNase inhibitor) was added. After incubation on ice for 30 min, the lysate was centrifuged at 12,000 × *g* at 4 °C for 10 min. An antibody and 30 μl of protein G magnetic beads were added to the lysate, which was subsequently incubated at 4 °C overnight. After washing with buffer solution (50 mM KCl, 25 mM Tris, 5 mM EDTA, 0.5 mM DTT, 0.5% NP-40), the coprecipitated RNA was extracted with TRIzol reagent, and rRNA was depleted by using an NEBNext rRNA Depletion Kit (New England Biolabs) prior to high-throughput sequencing on an Illumina NovaSeq platform.

### RNA bisulfite sequencing (RNA-BisSeq)

Cells were collected, and total RNA was extracted with TRIzol reagent. rRNA was depleted by using an NEBNext rRNA Depletion Kit (New England Biolabs), and then bisulfite conversion was performed according to the instructions of the EZ RNA Methylation Kit (Zymo Research). Converted RNA was collected, and a cDNA library was generated and sequenced.

### Chromatin immunoprecipitation (ChIP) and ChIP-seq

ChIP was performed by using the SimpleChIP Enzymatic Chromatin IP Kit (Cell Signaling Technology) according to the manufacturer’s instructions. Briefly, cells were subjected to crosslinking, and chromatin was isolated and digested with nuclease. The digested chromatin was incubated with an anti-E2F1 antibody (Thermo Fisher) for immunoprecipitation. Chromatin–protein complexes were isolated by protein G magnetic beads, crosslinking was reversed, and chromatin DNA was purified for PCR analysis or high-throughput sequencing on an Illumina NovaSeq platform.

### m^5^C RNA immunoprecipitation (m^5^C-RIP)

Cells were collected, and total RNA was extracted with TRIzol reagent. mRNA was purified with a PolyATtract mRNA Purification Kit (Promega) and fragmented with a MeRIP Kit (Millipore). Magnetic beads were incubated with 20 μl of protein G and an anti-m^5^C antibody (Abcam) at 4 °C for 4 h. Then, the anti-m^5^C antibody-coated protein G magnetic beads were added to the fragmented mRNA, and the mixture was incubated at 4 °C overnight. After washing with buffer solution (0.02% Triton X-100, 0.1% BSA) 3 times, the coprecipitated RNA was isolated with TRIzol reagent, and the target RNA expression was quantified via RT‒qPCR.

### Enhanced UV-crosslinking immunoprecipitation followed by PCR (eCLIP-PCR)

eCLIP was performed as previously described, with some modifications^29^. Cells were washed with ice-cold PBS and subjected to crosslinking by exposure to 254 nm ultraviolet light (150 mJ cm^−2^). The collected pellet was lysed with lysis buffer prior to partial RNase digestion. The lysate was incubated with the appropriate antibody overnight at 4 °C, after which protein G beads were added and the mixture was incubated for 2 h. After washing, the coprecipitated protein and RNA were harvested and analyzed by western blotting and PCR, respectively.

### RNA half-life assay

Cells in the control group and the knockdown group were seeded into 12-well plates. Then, 5 μg/ml actinomycin D (AbMole) was added to the cells when the confluence reached 70–80%. Cells were collected at the specified time points. Total RNA was extracted with TRIzol reagent and analyzed by RT‒qPCR.

### Luciferase reporter assay

The wild-type E2F1–3′UTR and the E2F1–3′UTR with mutation of the m^5^C sites were cloned and inserted into the pMirTarget vector. The wild-type *NSUN2* promoter (from −1000 bp with respect to the transcription start site with TGCGCGCGAAG) and its mutant (from −1000 bp with respect to the transcription start site with GGGATTCTTTG) were cloned and inserted into the pGL4.10 vector. The reporters and mutant constructs were cotransfected with the pRL-CMV plasmid as a luciferase control plasmid. After 48 h of transfection, Renilla and firefly luciferase activity was measured with a luciferase reporter assay kit (Promega).

### Polysome profiling

Cells were treated with 100 μg/mL cycloheximide (CHX; Merck Millipore, Germany) at 37 °C for 15 min and were then collected. After washing twice with cold PBS containing 100 μg/mL CHX, the cells were lysed on ice for 30 min with lysis buffer. The lysate was centrifuged, and the supernatant was collected. The supernatant was then ultracentrifuged in a 10–50% (w/v) sucrose gradient solution at 4 °C for 3 h at 38,000 × *g* (Beckman, XE-90 Ultracentrifuge). The sample was then fractionated using a Piston Gradient Fractionator (Biocomp) equipped with an EconoUV monitor (Bio-Rad) and a fraction collector (FC203B, Gilson). RNA and protein were isolated from each fraction for RT‒qPCR and western blot analysis, respectively.

### Analysis of sequencing data

Basic data analysis: FastQC version 0.11.9 was used for quality control for all deep sequencing samples. All the reads were mapped to the hg38 human genome assembly. For RNA-BisSeq, the adapters were trimmed with Cutadapt version 1.18, and the clean data were further analyzed with meRanTK version 1.2.0^30^. The fast meRanGs module with STAR version 2.6.1d was used for genome mapping^31^, and the meRanCall and meRanCompare modules were used to precisely identify the positions of m^5^C-modified and differentially methylated cytosines, respectively, with the default parameters. The RNA motif associated with m^5^C modification was searched with HOMER version 4.11^32^. m^5^C profiles along gene bodies were studied by using deepTools version 3.5.0^33^. For RNA-seq and RIP-seq data analysis, clean data were mapped to the genome by using STAR version 2.7.8a. All the reads for different genes were counted by using featureCounts version 2.0.3^34^. Differential gene expression was analyzed with the R package DESeq2^35^.

Integrative data analysis and statistics: Differentially methylated cytosines, which were unique to one of the datasets, were defined as m^5^C positions. Differentially expressed genes identified through RNA-seq were those for which |log2(shNSUN2/shNC)| > log2(1.5) and *p* < 0.05. The RIP targets were genes for which log2(IP/Input) > log2(1.5) and *p* < 0.05. Overexpression analysis was conducted by using the R Bioconductor package ClusterProfiler and the DAVID tool (https://david.ncifcrf.gov)^36^.

### Statistical analysis

The data are expressed as the means ± SDs. The significance of differences was evaluated using Student’s *t* test or an unpaired two-tailed *t* test. The Kaplan‒Meier method and the log-rank test were applied to estimate overall survival, progression-free survival, and their differences. Two-sided Wilcoxon and Mann‒Whitney tests were employed to compare the cumulative distribution functions of two sample sets. R v3.4.1 (https://www.r-project.org/) and Prism 9 (GraphPad Software, Inc.) were also used for statistical analysis. A *P* < 0.05 was considered to indicate a statistically significant difference.

## Results

### NSUN2 is overexpressed in ovarian cancer tissues, and NSUN2 overexpression is correlated with poor prognosis in patients with ovarian cancer

**Fig. 1:**
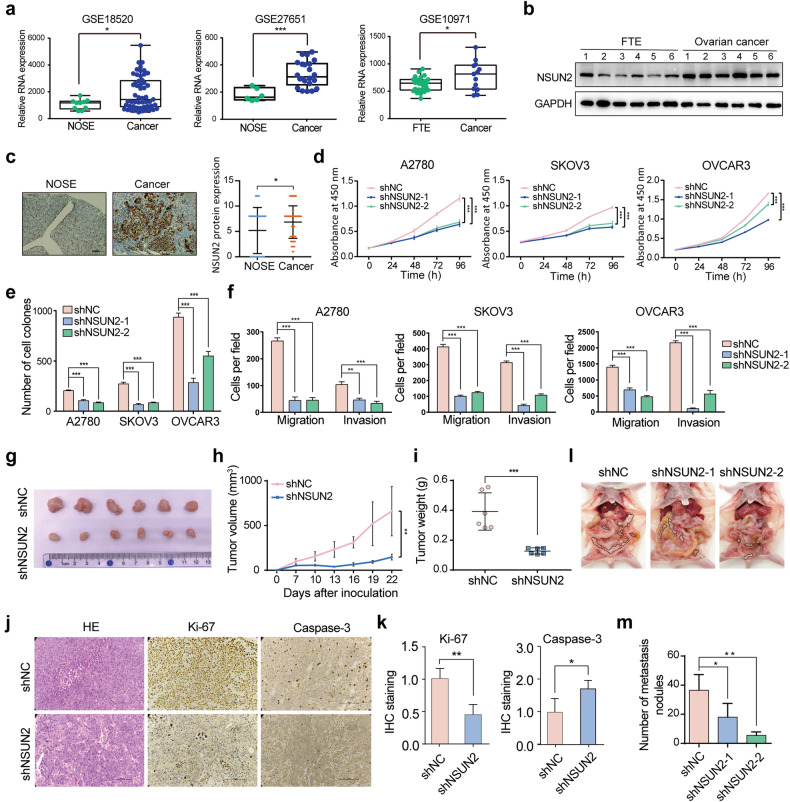
NSUN2 facilitates tumorigenesis and metastasis in ovarian cancer. **a** RNA expression of NSUN2 in ovarian cancer, according to GEO datasets (GSE19071, GSE18520, and GSE27651). **b** Western blot showing the protein level of NSUN2 in fresh ovarian cancer tissues and FTE tissues. **c** Relative NSUN2 protein expression was determined in ovarian surface epithelium and ovarian cancer specimens by immunohistochemistry, and representative immunohistochemical images are shown. Scale bar, 100 μm. **d** CCK-8 assays were used to evaluate the growth of ovarian cancer cells upon NSUN2 knockdown. **e** Colony formation assays showing the effect of NSUN2 knockdown on ovarian cancer cell growth. **f** Transwell migration and invasion assays of ovarian cancer cells with or without NSUN2 expression. **g**–**i** Tumorigenesis of ovarian cancer cells with or without NSUN2 expression was assessed by using nude mouse xenograft models. NSUN2-deficient and control OVCAR3 cells were subcutaneously injected into nude mice. The volume of the tumors formed was determined every 3 days (**h**). After 22 days, the tumors that formed were excised (**g**), and the tumor weights were measured (**i**). **j**, **k** Representative images and quantitative analysis of Ki-67 and Caspase-3 expression in xenograft sections. Scale bar, 100 μm. **l**, **m** Effect of NSUN2 knockdown on the metastasis of ovarian cancer cells and quantitative analysis of metastatic foci formed in the peritoneal cavity of mice. *p < 0.05, **p < 0.01, ***p < 0.001.

To explore the role of m^5^C modification in ovarian cancer, we analyzed the expression of 14 m^5^C regulators in the TCGA cohort. The results showed that multiple m^5^C regulators were dysregulated in ovarian cancer, among which the RNA methyltransferase NSUN2 exhibited the most dramatic alteration in expression, which was typically upregulated (Supplementary Fig. 1a). Similar results were also found in the TCGA pancancer cohort (Supplementary Fig. 1b). Genetic alteration analysis of the TCGA pancancer cohort revealed that *NSUN2* was amplified in various cancers, including ovarian cancer (Supplementary Fig. 1c, d). Moreover, the copy number of *NSUN2* was correlated with its expression level in ovarian cancer, indicating that the increased expression of NSUN2 in ovarian cancer is partially due to *NSUN2* gene amplification (Supplementary Fig. 1e, f). According to the Oncomine database, NSUN2 was also amplified and upregulated in ovarian cancer tissues compared with normal ovarian surface epithelium (NOSE) tissues (Supplementary Fig. 1g, h). Consistently, according to several GEO ovarian cancer datasets, NSUN2 expression was aberrantly increased in ovarian cancer tissues compared with NOSE or fallopian tube epithelium (FTE) tissues (Fig. 1a). We subsequently collected fresh ovarian cancer tissues and FTE tissues to assess the protein expression of NSUN2 in ovarian cancer. NSUN2 protein expression was elevated in fresh ovarian cancer tissues compared with FTE tissues, and similar results were observed in two independent ovarian cancer cohorts in which protein expression in ovarian cancer and FTE tissues was evaluated (Fig. 1b, Supplementary Fig. 1i)^37,38^. Furthermore, we constructed a tissue microarray containing 27 NOSE and 134 ovarian cancer specimens and found that NSUN2 protein expression was significantly upregulated in ovarian cancer tissues compared with NOSE tissues (Fig. 1c). These data indicate that NSUN2 is overexpressed in ovarian cancer.

Despite the lack of significant differences in the NSUN2 RNA level among different grades of serous ovarian cancer, NSUN2 protein expression was increased in high-grade (grade 2 and grade 3) and advanced-stage ovarian cancer specimens (Supplementary Fig. 1j, k). Furthermore, Kaplan‒Meier analysis revealed that increased expression of NSUN2 was strongly correlated with poor prognosis, as indicated by the results of overall survival and progression-free survival analyses of ovarian cancer patients (Supplementary Fig. 1l). Collectively, these results support a potential tumor-promoting role for NSUN2 in ovarian cancer.

### NSUN2 promotes tumorigenesis and metastasis in ovarian cancer

To determine the tumorigenic function of NSUN2 in ovarian cancer, we performed gain- and loss-of-function assays in ovarian cancer cells. First, we overexpressed NSUN2 in ovarian cancer cells, namely, A2780, OVCAR3, and SKOV3 cells (Supplementary Fig. 2a). CCK-8 and colony formation assays revealed that forced expression of NSUN2 significantly promoted cell growth (Supplementary Fig. 2b–d). The Transwell assay revealed that NSUN2 overexpression promoted the migration and invasion of these ovarian cancer cells (Supplementary Fig. 2e, f). The subcutaneous tumorigenesis assay showed that NSUN2 overexpression facilitated tumor growth from ovarian cancer cells (Supplementary Fig. 2g–i). Then, we depleted NSUN2 in ovarian cancer cells by using shRNAs (Supplementary Fig. 2j). Loss of NSUN2 strongly inhibited the growth, migration, and invasion of ovarian cancer cells (Fig. 1d–f, Supplementary Fig. 2k, l). Additionally, a subcutaneous tumorigenesis model and a peritoneal metastasis model were used to assess the oncogenic role of NSUN2 in ovarian cancer in vivo. As expected, NSUN2 knockdown delayed the progression of OVCAR3 xenograft tumors, as the weight and volume of the NSUN2-defective tumors were significantly decreased compared with those of the NSUN2-competent tumors (Fig. 1g–i). Immunohistochemical (IHC) staining for Ki-67 and Caspase-3 showed that tumors from the NSUN2 knockdown group exhibited impaired cellular proliferation (Fig. 1j, k). To examine the effect of NSUN2 on the ability of cells to form secondary tumors in the peritoneal cavity, we injected NSUN2-defective and control ovarian cancer cells into the peritoneal cavity. NSUN2 deficiency prevented the formation of metastatic foci by ovarian cancer cells in the peritoneal cavity (Fig. 1l, m). Taken together, these results demonstrate the oncogenic role of NSUN2 in ovarian cancer through the regulation of cell growth and metastasis.

### NSUN2 promotes m^5^C RNA modification in ovarian cancer cells

**Fig. 2:**
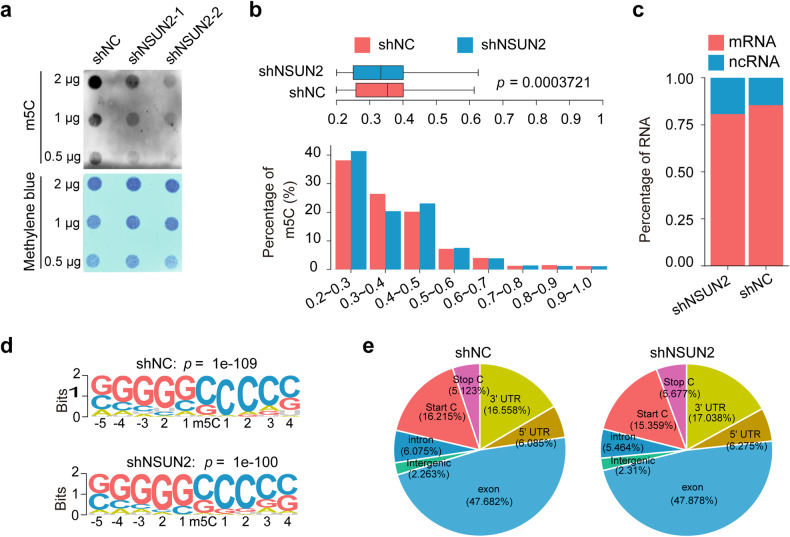
NSUN2 mediates mRNA m^5^C modification in ovarian cancer cells. **a** Dot blot showing the global m^5^C modification of mRNAs in ovarian cancer cells upon NSUN2 knockdown. **b** Histogram and box plot displaying the mRNA m^5^C levels in ovarian cancer cells with or without NSUN2 overexpression. **c** Percentages of transcript species with m^5^C sites. **d** Logo plot of the sequences adjacent to mRNA m^5^C sites identified through HOMER motif analysis. *p* values were calculated by the HOMER algorithm. **e** Transcriptome-wide distribution of hypermethylated m^5^C peaks in ovarian cancer cells with or without NSUN2 overexpression. Pie chart presenting the proportions of m^5^C sites within distinct mRNA regions: CDS, intron, 5′-UTR, 3′-UTR, start codon, and stop codon.

Given that NSUN2 has been demonstrated to catalyze m^5^C modification of mRNA^21^, we first examined the localization of NSUN2 in ovarian cancer cells by immunofluorescence staining. NSUN2 was mainly distributed in the nucleus, and NSUN2 knockdown decreased its protein expression (Supplementary Fig. 3a). A dot blot assay was subsequently performed using an antibody specific for m^5^C-modified RNA to confirm whether NSUN2 regulates global mRNA m^5^C modification. We found that knockdown of NSUN2 reduced m^5^C deposition on mRNA in both A2780 and OVCAR3 ovarian cancer cells (Fig. 2a, Supplementary Fig. 3b). Conversely, NSUN2 overexpression increased mRNA m^5^C modification in ovarian cancer cells (Supplementary Fig. 3c). To determine the specific mRNA m^5^C modification mediated by NSUN2, we mapped the transcriptome-wide alterations in m^5^C modification at single-base resolution in ovarian cancer cells following NSUN2 knockdown via RNA-BisSeq. m^5^C modification at 16,536 sites within 6,192 RNAs was reduced upon NSUN2 knockdown, and these m5C-modified sites were identified as m^5^C hypermethylated sites in ovarian cancer cells. The global m^5^C modification level was also decreased upon NSUN2 knockdown (Fig. 2b). The median methylation percentage of the m^5^C sites in ovarian cancer cells was approximately 30%, and the m^5^C percentage in the majority of transcripts was lower than 40%. Approximately 80% of the transcripts with m^5^C modifications were mRNAs (Fig. 2c). In line with the findings of a previous study^21^, m^5^C sites were enriched in CG-rich sequences, as shown in the sequence logo (Fig. 2d). The most enriched m^5^C sites were in exons, although other regions, including untranslated regions (UTRs) and translation initiation sites (start C), were also subjected to m^5^C modification (Fig. 2e, Supplementary Fig. 3d). Notably, m^5^C sites tended to be distributed in the 5′-UTRs and start codons of mRNA transcripts, consistent with the findings in mouse embryonic stem cells and the brain, but were enriched at the 5′ and 3′ ends of noncoding RNA transcripts. No significant difference in the distribution profile of m^5^C sites in mRNA transcripts was found between control and NSUN2-deficient ovarian cancer cells (Supplementary Fig. 3d). Gene Ontology (GO) analysis of transcripts with m^5^C hypermethylation in ovarian cancer cells revealed that tumor-associated regulatory pathways were enriched in these transcripts (Supplementary Fig. 3e).

### A multiomics strategy reveals the targets of NSUN2 in ovarian cancer

**Fig. 3:**
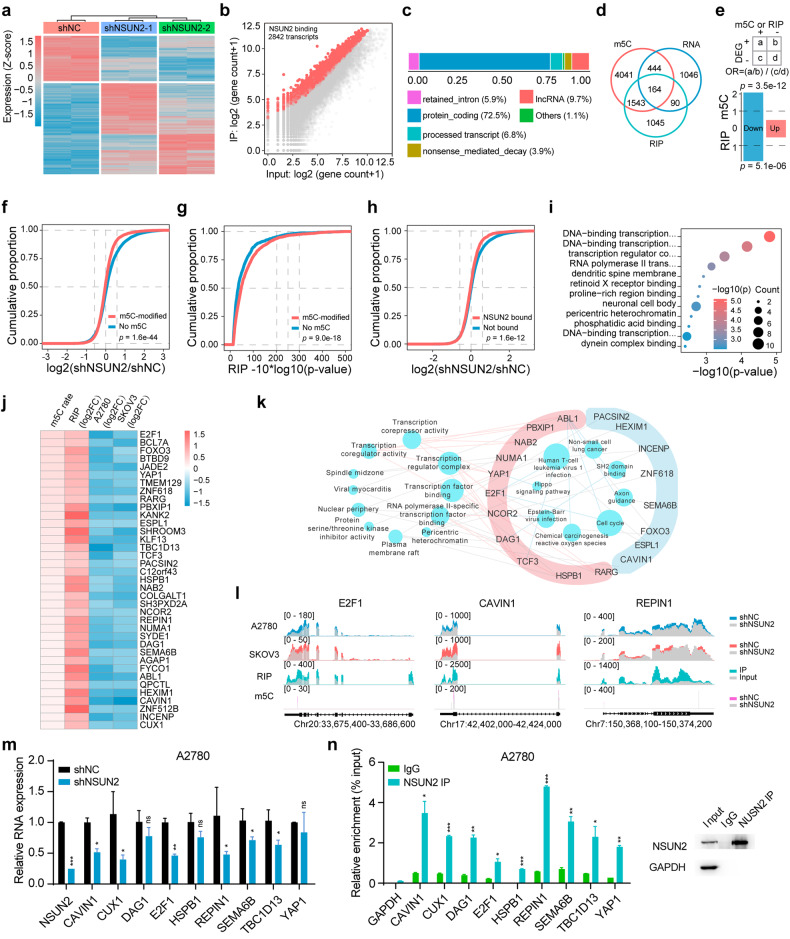
Transcriptome-wide identification of NSUN2 targets. **a** Heatmap showing the differentially expressed genes (DEGs) in NSUN2-knockdown compared with control A2780 cells. **b** Dot plot displaying NSUN2-binding transcripts. After RIP-seq of NSUN2, the gene counts in both the input group and IP group were evaluated via dot blotting. The red dots represent the genes enriched in the IP group as NSUN2-binding transcripts. **c** Analysis of NSUN2-binding transcript species. LncRNA: long noncoding RNA. **d** Venn diagram showing the overlap of RNAs identified via RNA-BisSeq (m^5^C), RNA-seq (RNA) and NSUN2 RIP-seq (RIP). **e** Upper panel: formula for calculating the odds ratio. Lower panel: odds ratios of DEGs upon NSUN2 knockdown for NSUN2-binding versus non-NSUN2-binding transcripts as well as for transcripts with hypermethylated m^5^C peaks. The *p* value was calculated by two-tailed Fisher’s exact test. **f** Cumulative curves showing hypermethylated m^5^C peaks in ovarian cancer cells after NSUN2 silencing. **g** Cumulative curves showing the effect of hypermethylated m^5^C peaks in ovarian cancer cells on NSUN2 binding. **h** Cumulative curves revealing NSUN2-binding transcripts in ovarian cancer cells after NSUN2 silencing. *p* values were calculated using two-sided Wilcoxon and Mann‒Whitney tests. **i** GO functional enrichment analysis of 81 downregulated genes in NSUN2-knockdown cells. **j** Heatmap showing the crucial targets of NSUN2 in ovarian cancer cells according to the results of integrated multiomics analysis. **k** Functional annotations of the 37 crucial targets of NSUN2 based on GO analysis. The pink color of the outer ring indicates transcription-associated terms. **l** Metagene plot showing the distribution of m^5^C sites and RNA abundances in ovarian cancer cells upon NSUN2 knockdown as well as upon NSUN2 binding. **m** RT‒qPCR was used to measure the RNA levels of NSUN2 targets upon NSUN2 knockdown in A2780 cells. **n** RIP-PCR was used to evaluate the interactions between NSUN2 and its target transcripts. Proteins immunoprecipitated with an antibody specific for NSUN2 were analyzed via western blotting. *p < 0.05, **p < 0.01, ***p < 0.001; ns, not significant.

To further explore NSUN2-mediated regulation of gene expression, RNA-seq was performed on NSUN2-deficient ovarian cancer cells, and RIP-seq was performed on ovarian cancer cells. In ovarian cancer cells, NSUN2 knockdown resulted in altered expression of 1744 genes, and RIP-seq revealed that NSUN2 could bind to 2842 transcripts (Fig. 3a, b; Supplementary Fig. 4a). The transcripts identified via RNA-seq and RIP-seq were enriched in various signaling pathways, according to GO analysis (Supplementary Fig. 4b, c). Approximately 72.5% of the NSUN2-bound transcripts were protein-coding RNAs (Fig. 3c). Through integrated analysis of RNA-seq, RIP-seq and RNA-BisSeq data, 163 transcripts were identified as potential targets of NSUN2 (Fig. 3d). To further pinpoint the mechanism of gene regulation mediated by NSUN2, we used the odds ratio (OR) to evaluate the correlations between the m^5^C-hypermethylated RNAs or NSUN2-bound transcripts and the differentially expressed genes (DEGs) in NSUN2-deficient ovarian cancer cells. We found that both m^5^C-modified RNAs and NSUN2-bound RNAs were preferentially downregulated upon NSUN2 knockdown, suggesting that NSUN2 positively regulates the expression of its targets (Fig. 3e). Consistently, the m^5^C-hypermethylated RNAs in ovarian cancer cells tended to be downregulated upon NSUN2 knockdown, as determined by cumulative analysis (Fig. 3f). RNAs for which NSUN2 had a high binding affinity also exhibited m^5^C hypermethylation and decreased expression in NSUN2-deficient cells (Fig. 3g, h). GO analysis revealed that transcriptional regulation-associated pathways were enriched in 81 downregulated genes among the 163 candidate NSUN2 targets (Fig. 3i). Thus, these data demonstrated that NSUN2 could promote m^5^C modification of mRNAs and positively regulate their expression.

To narrow the field of NSUN2 targets in ovarian cancer cells, RNA-seq was also performed in SKOV3 cells following NSUN2 knockdown, and bioinformatics analysis revealed that 1,748 genes were significantly modulated by NSUN2 knockdown (Supplementary Fig. 4d). A moderate correlation between the DEGs was found in these ovarian cancer cell lines, and the DEGs also overlapped with the transcripts with m^5^C hypermethylation and NSUN2 binding (Supplementary Fig. 4e, f). Integrated analysis of the 726 downregulated transcripts in SKOV3 cells, the downregulated transcripts occupied by NSUN2, and the m^5^C-modified transcripts in A2780 cells identified 37 genes as the crucial targets of NSUN2 (Fig. 3j). Functional annotation of these target genes revealed that mainly transcription-associated terms were enriched in these genes, in agreement with the above analysis of the targets’ function in A2780 ovarian cancer cells (Fig. 3i, k). Among these candidates, we selected a set of genes associated with tumorigenesis to validate the regulatory effect of NSUN2. The m^5^C sites in each RNA sample determined by RNA-BisSeq and RNA expression profiles determined by RNA-seq are displayed in Fig. 3l and Supplementary Fig. 4g. RT‒qPCR and RIP‒PCR assays confirmed the changes in the expression of the target genes upon NSUN2 knockdown and the interactions of NSUN2 with these genes (Fig. 3m, n; Supplementary Fig. 4h‒k).

### NSUN2 and YBX1 regulate the stability of E2F1 mRNA in an m^5^C-dependent manner

**Fig. 4:**
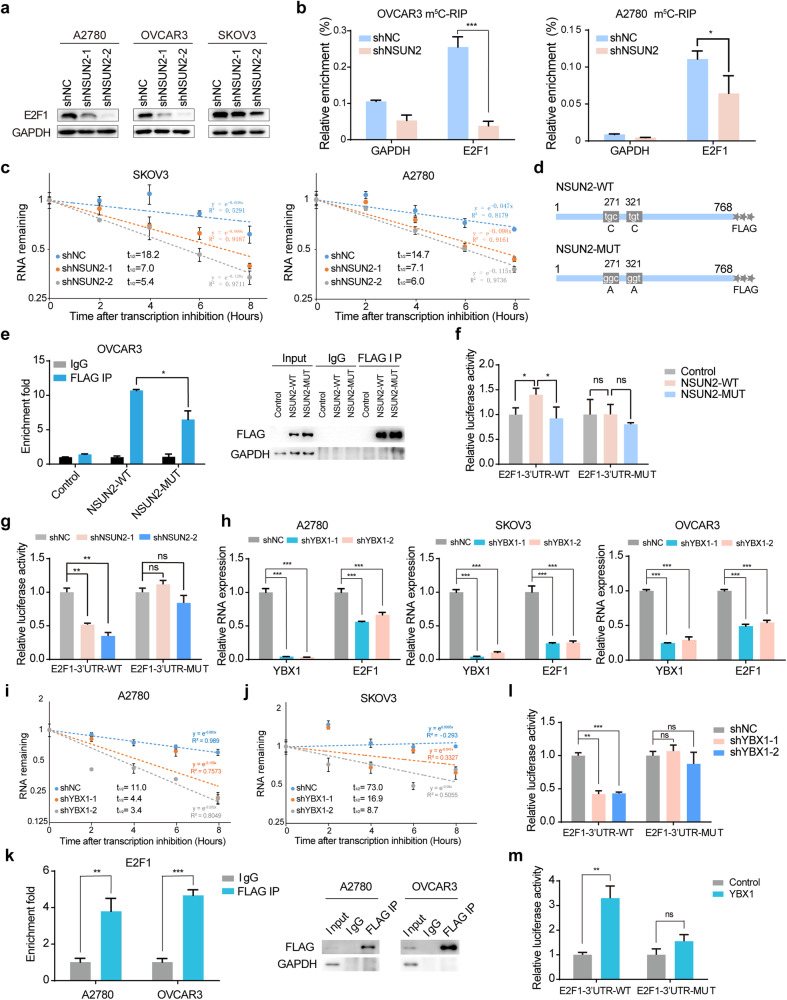
E2F1 expression is regulated by NSUN2 and YBX1 in an m^5^C-dependent manner. **a** E2F1 protein expression in ovarian cancer cells after NSUN2 knockdown was measured via western blotting. **b** m^5^C-RIP followed by RT‒qPCR confirmed that NSUN2 mediated the m^5^C modification of E2F1 mRNA. **c** The half-life of E2F1 mRNA was evaluated in ovarian cancer cells upon NSUN2 knockdown. **d** Schematic of wild-type NSUN2 and catalytic activity-deficient NSUN2 tagged with FLAG. **e** RIP-PCR was performed to confirm the interactions of NSUN2 and the NSUN2 mutant with E2F1 mRNA. Proteins immunoprecipitated with an antibody specific for FLAG were analyzed via western blotting. **f** The relative firefly luciferase activity of the reporters carrying the wild-type E2F1 3′-UTR or the E2F1 3′-UTR with m^5^C site deletion in cells coexpressing NSUN2 or the NSUN2 mutant was measured and normalized to Renilla luciferase activity. **g** The relative firefly luciferase activity of the reporters carrying the wild-type E2F1 3′-UTR or the E2F1 3′-UTR with m^5^C site deletion in control and NSUN2-deficient ovarian cancer cells was measured and normalized to Renilla luciferase activity. **h** E2F1 expression was measured in ovarian cancer cells upon YBX1 depletion. **i** Effect of YBX1 knockdown on the half-life of E2F1 mRNA in ovarian cancer cells. **j** Effect of YBX1 knockdown on the half-life of E2F1 mRNA in SKOV3 cells. **k** The results of RIP-PCR confirmed that YBX1 interacted with E2F1 mRNA. Proteins immunoprecipitated with an antibody specific for FLAG were analyzed via western blotting. **l** The relative firefly luciferase activity of the reporters carrying the wild-type E2F1 3′-UTR or the E2F1 3′-UTR with m^5^C site deletion in control and YBX1-deficient ovarian cancer cells was measured and normalized to Renilla luciferase activity. **m** The relative luciferase activity of the reporters carrying the wild-type E2F1 3′-UTR or the E2F1 3′-UTR with m^5^C site deletion in HEK293T cells coexpressing YBX1 was measured and normalized to Renilla luciferase activity. *p < 0.05, **p < 0.01, ***p < 0.001.

The GO analysis above revealed that DNA-binding transcription factors were enriched among the potential targets of NSUN2 (Fig. 3i, k), and we found that the expression of the oncogenic transcription factor E2F1, which had the highest level of m^5^C modification, was significantly decreased in NSUN2-deficient ovarian cancer cells. To determine the regulatory effect of NSUN2 on E2F1, we measured E2F1 protein expression upon NSUN2 knockdown in ovarian cancer cells. Western blot analysis revealed that NSUN2 knockdown significantly reduced the E2F1 protein abundance (Fig. 4a). To determine the underlying mechanism, we performed an m^5^C-RIP assay to determine whether NSUN2 mediates the m^5^C modification of E2F1 mRNA. In line with the RNA-BisSeq results, E2F1 mRNA was found to undergo m^5^C modification, which was significantly decreased upon NSUN2 knockdown (Fig. 4b). We also assessed the effect of NSUN2 on E2F1 mRNA stability and found that NSUN2 depletion reduced the half-life of E2F1 mRNA in ovarian cancer cells (Fig. 4c, Supplementary Fig. 5a), suggesting that NSUN2 promoted m^5^C modification of E2F1 mRNA and facilitated its stability. We next mutated the catalytic sites (C271A and C321A) in NSUN2 (NSUN2-WT)^22^ and transfected the resulting constructs into ovarian cancer cells. The related RIP assays showed that, compared with NSUN2-WT, the NSUN2 mutant (NSUN2-MUT) exhibited a reduced but not completely abolished binding affinity for E2F1 mRNA (Fig. 4d, e; Supplementary Fig. 5b, c). NSUN2-WT promoted E2F1 expression, but NSUN2-MUT failed to upregulate E2F1 expression (Supplementary Fig. 5d). In agreement with the mapping of the m^5^C site in the 3′-UTR of E2F1 mRNA by RNA-BisSeq, the eCLIP-PCR assay showed that NSUN2 preferentially bound to the 3′-UTR of E2F1 mRNA in the vicinity of its m^5^C modification site (Supplementary Fig. 5e). To further confirm that the ability of NSUN2 to modulate E2F1 expression relies on m^5^C modification, luciferase reporter vectors with wild-type and m^5^C site-mutated E2F1 3′-UTRs were constructed (Supplementary Fig. 5f). Subsequently, luciferase activity was measured. We found that wild-type NSUN2 increased the luciferase activity of the wild-type E2F1 3′-UTR (E2F1–3′-UTR-WT) but not the m^5^C site-mutated E2F1 3′-UTR (E2F1–3′-UTR-MUT), whereas the NSUN2 mutant failed to increase the luciferase activity of either the wild-type E2F1 3′-UTR or the m^5^C site-mutated E2F1 3′-UTR (Fig. 4f). Moreover, NSUN2 knockdown substantially decreased the luciferase activity of the wild-type E2F1 3′-UTR but not the m^5^C site-mutated E2F1 3′-UTR (Fig. 4g), highlighting that the regulation of E2F1 expression by NSUN2 is dependent on m^5^C modification.

To elucidate how m^5^C modification affects the stability of E2F1 mRNA, we investigated the effects of ALYREF, Lin28B and YBX1 on E2F1 expression, as these proteins were demonstrated to function as m^5^C readers and regulate the expression of m^5^C-modified RNAs^21,22,26^. Thus, we knocked down these m^5^C readers separately in ovarian cancer cells and evaluated the changes in E2F1 expression. We found that YBX1 knockdown but not ALYREF or Lin28B knockdown led to a decreased RNA level of E2F1 (Fig. 4h, Supplementary Fig. 5g). Western blot analysis revealed that YBX1 knockdown decreased the protein expression of E2F1 in ovarian cancer cells, and half-life assays showed that YBX1 increased the stability of E2F1 mRNA (Fig. 4i, j; Supplementary Fig. 5h, i). RIP assays also demonstrated that YBX1 can bind to E2F1 mRNA (Fig. 4k). Additionally, YBX1 knockdown caused a decrease in the luciferase activity of the wild-type E2F1 3′-UTR but not the m^5^C site-mutated E2F1 3′-UTR (Fig. 4l), suggesting that m^5^C modification is indispensable for E2F1 regulation by YBX1. In contrast, overexpression of YBX1 promoted the luciferase activity of the wild-type E2F1 3′-UTR but not the m^5^C site-mutated E2F1 3′-UTR (Fig. 4m). To further confirm that NSUN2-mediated m^5^C modification regulates E2F1 expression in a manner dependent on YBX1, we overexpressed NSUN2 in ovarian cancer cells with or without YBX1 expression and assessed the expression of E2F1. YBX1 depletion disrupted the effect of NSUN2 overexpression on E2F1 cells (Supplementary Fig. 5j). Consistently, NSUN2 overexpression failed to increase the luciferase activity of the wild-type E2F1 3′-UTR in YBX1-deficient cells, but restoration of YBX1 expression rescued the luciferase activity of the wild-type E2F1 3′-UTR induced by NSUN2 overexpression, suggesting that YBX1 is involved in the regulation of E2F1 expression by NSUN2 (Supplementary Fig. 5k). Taken together, these results indicate that NSUN2 and YBX1 upregulate E2F1 expression in an m^5^C-dependent manner.

### YBX1 undergoes phase separation and is upregulated in ovarian cancer

**Fig. 5:**
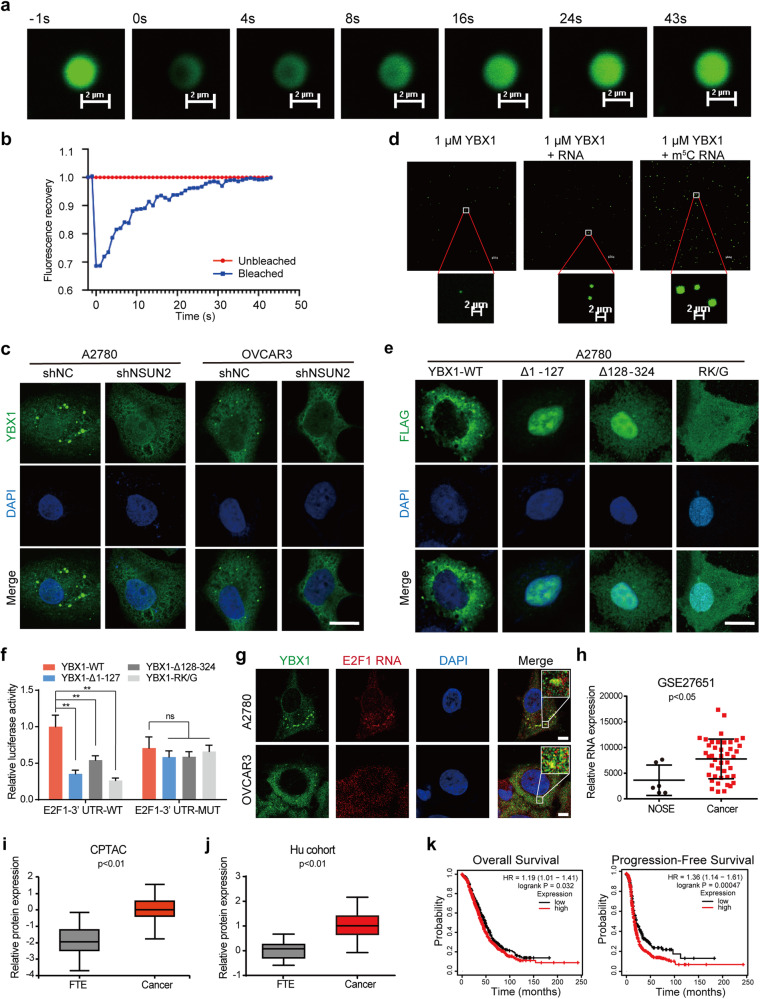
YBX1 condensation regulates E2F1 expression. **a** Representative images of fluorescence recovery. Scale bar, 2 μm. **b** Quantification of the FRAP assay results shown in (**a**). **c** Immunofluorescence imaging of YBX1 revealed YBX puncta in ovarian cancer cells upon NSUN2 knockdown. Scale bar, 10 μm. **d** Phase separation of the YBX1 protein with m^5^C-modified RNA or non-m^5^C-modified RNA. **e** Immunofluorescence imaging of GFP-fused YBX1 and different YBX1 mutants. Scale bar, 10 μm. **f** The relative luciferase activity of the reporter carrying the wild-type E2F1 3′-UTR or the E2F1 3′-UTR with m^5^C site deletion in HEK293T cells coexpressing YBX1 or its mutants was measured and normalized to Renilla luciferase activity. **g** RNA FISH combined with IF was used to detect the colocalization of E2F1 mRNA and YBX1 condensates. **h** RNA expression of YBX1 in ovarian cancer samples, according to the GSE27651 dataset. **i** Protein expression of YBX1 in ovarian cancer tissues and FTE tissues in the CPTAC database. **j** Protein expression of YBX1 in ovarian cancer and FTE tissues in the Hu cohort. **k** Kaplan‒Meier analysis was performed to evaluate the associations of YBX1 expression with overall survival and progression-free survival in patients with ovarian cancer. **p < 0.01, ns not significant.

Multiple RNA-binding proteins undergo liquid‒liquid phase separation, thus forming a liquid‒like droplet and regulating the fate of target RNAs^39–42^. To further explore how YBX1 regulates m^5^C-modified RNAs, we examined the extent of YBX1 condensation in ovarian cancer cells. We expressed the full-length recombinant YBX1 protein fused to GFP in *E. coli* cells and performed phase separation assays with the purified protein in vitro. Consistent with the findings of previous studies^40^, at increasing protein concentrations, YBX1 underwent phase separation, and two pure YBX1 droplets combined to form a larger droplet (Supplementary Fig. 6a, b). Fluorescence recovery after photobleaching (FRAP) assays also demonstrated that the fluorescence signal of the YBX1-GFP protein was recovered after bleaching (Fig. 5a, b). Then, we performed immunofluorescence staining with an antibody specific for YBX1 to confirm YBX1 condensation in ovarian cancer cells and found that YBX1 was concentrated in puncta in all three ovarian cancer cell lines (Supplementary Fig. 6c). To determine the effect of m^5^C modification on YBX1 phase separation, we evaluated changes in the number of YBX1 puncta upon NSUN2 knockdown. Interestingly, NSUN2 knockdown significantly disrupted the formation of YBX1 puncta (Fig. 5c). In vitro phase separation assays also showed that m^5^C-modified RNA but not non-m^5^C-modified RNA could enhance the phase separation of YBX1 (Fig. 5d). To assess whether YBX1 condensation is involved in E2F1 regulation, we first constructed three YBX1 mutants, namely, two deletion mutants (Δ1–127 and Δ128–324) and a point mutant (RK/G), as demonstrated in a previous study^40^. Consistently, both YBX1-Δ128–324 and YBX1-RK/G lost phase separation ability, and YBX1-Δ1–127 phase separated in the nucleus (Fig. 5e). Luciferase assays demonstrated that YBX1-Δ128–324 and YBX1-RK/G could not promote the luciferase activity of the wild-type E2F1 3′-UTR or the m^5^C site-mutated E2F1 3′-UTR (Fig. 5f). Notably, RNA FISH assays showed that E2F1 mRNA was distributed in cytoplasmic YBX1 condensates (Fig. 5g). We also performed polysome profiling assays in ovarian cancer cells to examine the distribution of the YBX1 protein and E2F1 mRNA in the translationally active and translationally inactive fractions. The results showed that both the YBX1 protein and E2F1 mRNA were distributed in polysome fractions, which represent active translation, although YBX1 was also observed in the translationally inactive fractions (Supplementary Fig. 6d, e). These results suggest that YBX1 phase separation regulates E2F1 expression.

### YBX1 and E2F1 function as tumor promoters in ovarian cancer

Considering that YBX1 and NSUN2 regulate E2F1 expression in ovarian cancer, we examined the role of YBX1 in ovarian cancer. Like NSUN2, YBX1 was amplified in multiple cancers, including ovarian cancer (Supplementary Fig. 7a, b). Gene amplification contributed to YBX1 mRNA upregulation followed by increased YBX1 protein expression (Fig. 5h–j, Supplementary Fig. 7c–i). High expression of YBX1 predicted poor prognosis in ovarian cancer patients (Fig. 5k). YBX1 knockdown significantly suppressed tumorigenesis and metastasis in vitro and in vivo (Supplementary Fig. 8a–h).

E2F1 has been implicated in the malignancy of several cancers^43,44^; thus, we sought to characterize its role in ovarian cancer. E2F1 was knocked down in ovarian cancer cells, and CCK-8 assays were then performed (Supplementary Fig. 9a). We found that E2F1 deficiency dramatically impeded ovarian cancer cell growth. The colony formation assay showed that the colony numbers in all three ovarian cancer cell line groups were substantially decreased upon E2F1 knockdown (Supplementary Fig. 9b-d). In vitro Transwell assays also revealed that E2F1 inhibition significantly diminished the migration and invasion abilities of ovarian cancer cells (Supplementary Fig. 9e, f). A subcutaneous tumorigenesis model and peritoneal metastasis model were also used to investigate the oncogenic role of E2F1 in ovarian cancer in vivo. The subcutaneous tumorigenesis assay revealed that E2F1 knockdown reduced the volume and weight of tumors formed from ovarian cancer cells (Supplementary Fig. 9g-i), which phenocopied the effect of NSUN2 deficiency. Accordingly, E2F1 knockdown attenuated the metastasis of ovarian cancer cells to the peritoneal cavity (Supplementary Fig. 9j, k). Taken together, these data indicate the crucial function of E2F1 in ovarian cancer progression.

### E2F1 transcriptionally regulates NSUN2 expression

**Fig. 6:**
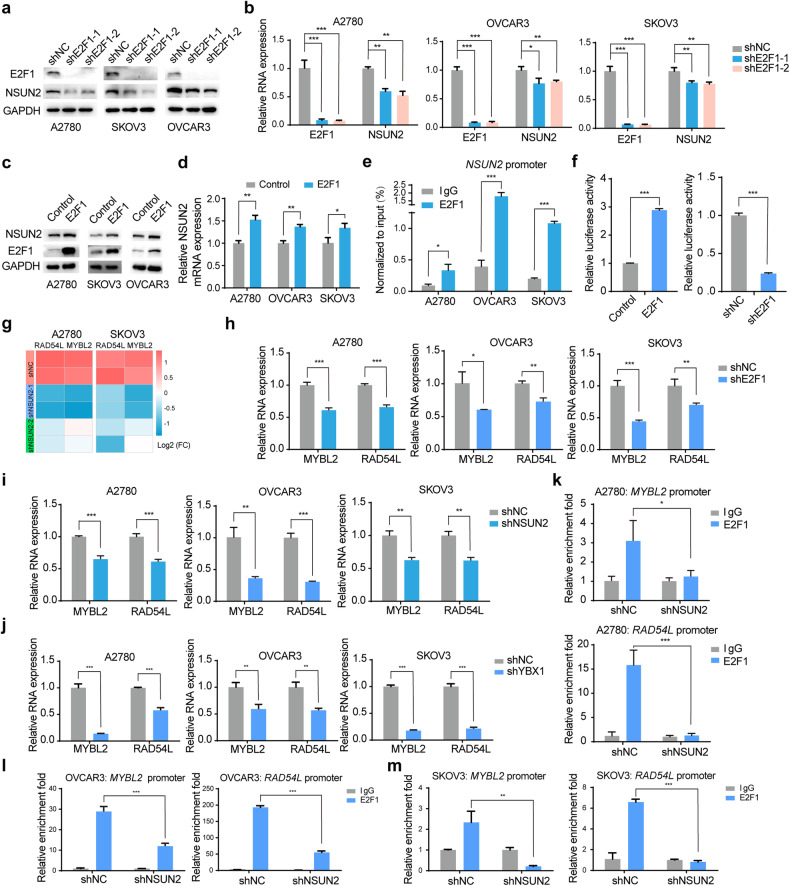
E2F1 transcriptionally regulates NSUN2 expression. **a** The NSUN2 protein abundance was measured in ovarian cancer cells upon E2F1 depletion. **b** The NSUN2 RNA level was measured in ovarian cancer cells upon E2F1 depletion. **c** NSUN2 protein expression was measured in ovarian cancer cells following E2F1 overexpression. **d** The NSUN2 RNA level was measured in ovarian cancer cells upon E2F1 overexpression. **e** The results of ChIP assays confirmed the interaction between E2F1 and the *NSUN2* promoter in ovarian cancer cells. **f** The relative luciferase activity of the reporter carrying the *NSUN2* promoter in HEK293T and SKOV3 cells with E2F1 overexpression or knockdown was measured and normalized to Renilla luciferase activity. **g** Heatmap showing the changes in MYBL2 and RAD54L expression in ovarian cancer cells upon NSUN2 knockdown. **h**, **i** MYBL2 and RAD54L RNA levels were validated in ovarian cancer cells upon loss of E2F1 or NSUN2 expression. **j** YBX1 regulated the expression of MYBL2 and RAD54L in ovarian cancer cells. **k** ChIP assays of E2F1 were performed in control and NSUN2-deficient A2780 ovarian cancer cells to test whether NSUN2 affects the enrichment of E2F1 at the promoters of MYBL2 and RAD54L. **l**, **m** ChIP assays of E2F1 were performed in control and NSUN2-deficient OVCAR3 and SKOV3 cells to test whether NSUN2 affects the binding of E2F1 to the promoters of MYBL2 and RAD54L. *p < 0.05, **p < 0.01, ***p < 0.001.

Intriguingly, we found that NSUN2 protein expression decreased following E2F1 knockdown when we investigated the function of E2F1 in ovarian cancer cells (Fig. 6a). Considering that E2F1 functions as a transcription factor, we speculated that E2F1 regulates NSUN2 expression at the transcriptional level. To verify this hypothesis, we monitored the mRNA abundance of NSUN2 upon E2F1 knockdown and observed that E2F1 knockdown reduced the mRNA level of NSUN2 (Fig. 6b). Conversely, forced expression of E2F1 upregulated NSUN2 expression at both the transcriptional and translational levels (Fig. 6c, d), consistent with the findings of a previous study showing that E2F1 regulates NSUN2 transcription in esophageal squamous cell carcinoma^26^. To further confirm this, we conducted a ChIP assay by using an antibody specific for E2F1 and found that E2F1 could bind to the *NSUN2* promoter (Fig. 6e). We constructed a luciferase reporter vector containing the *NSUN2* promoter, and luciferase activity assays showed that E2F1 could induce the luciferase activity of the *NSUN2* promoter (Fig. 6f). In contrast, E2F1 knockdown reduced the luciferase activity of the *NSUN2* promoter (Fig. 6f). We subsequently analyzed the promoter of *NSUN2* and found that it contains one potential E2F1 binding site. Thus, we constructed a luciferase reporter vector with mutation of the E2F1 binding site in the *NSUN2* promoter, and luciferase activity assays revealed that mutation of the E2F1 binding site in the *NSUN2* promoter significantly decreased luciferase activity compared with that of the wild-type NSUN2 promoter (Supplementary Fig. 10a). These data suggest that in addition to gene amplification, E2F1-mediated transcriptional activation also contributes to the upregulation of NSUN2 in ovarian cancer.

We next sought to verify the other targets transcriptionally regulated by NSUN2 in ovarian cancer. Toward this end, we performed E2F1 ChIP-seq in ovarian cancer cells and identified 1804 binding sites within 1733 genes (Supplementary Fig. 10b). We integrated the E2F1 ChIP-seq and RNA-seq data for NSUN2 in ovarian cancer cells and found that 120 genes were potential targets of both NSUN2 and E2F1 (Supplementary Fig. 10b). Among these potential targets, two oncogenes, MYBL2 and RAD54L, which were previously demonstrated to be targets of E2F1^45–49^, were downregulated in both cancer cell lines upon NSUN2 knockdown (Fig. 6g). The IGV plot shows the binding of E2F1 to the promoters of MYBL2 and RAD54L (Supplementary Fig. 10c). Moreover, the *NSUN2* promoter was also bound by E2F1 (Supplementary Fig. 10c). An E2F1 ChIP assay was performed to confirm that E2F1 binds to the promoters of *MYBL2* and *RAD54L* (Supplementary Fig. 10d). Then, we detected the expression of these two genes upon NSUN2 or E2F1 knockdown. Knockdown of either NSUN2 or E2F1 resulted in decreased expression of MYBL2 and RAD54L (Fig. 6h, i). Accordingly, YBX1 knockdown also decreased the expression of these two targets (Fig. 6j). We presumed that NSUN2 might modulate the expression of MYBL2 and RAD54L through E2F1-mediated transcriptional control. To confirm this, we further performed E2F1 ChIP assays in ovarian cancer cells following NSUN2 knockdown. Interestingly, NSUN2 knockdown significantly attenuated E2F1 enrichment at the promoters of *MYBL2* and *RAD54L* (Fig. 6k–m). Consistent with these findings, YBX1 knockdown also decreased the binding of E2F1 to the promoters of *MYBL2* and *RAD54L* (Supplementary Fig. 10e). Thus, these data suggest that E2F1 could regulate the transcription of NSUN2 as well as that of MYBL2 and RAD54L in ovarian cancer cells.

### E2F1 is closely involved in the tumor-promoting function of NSUN2

**Fig. 7:**
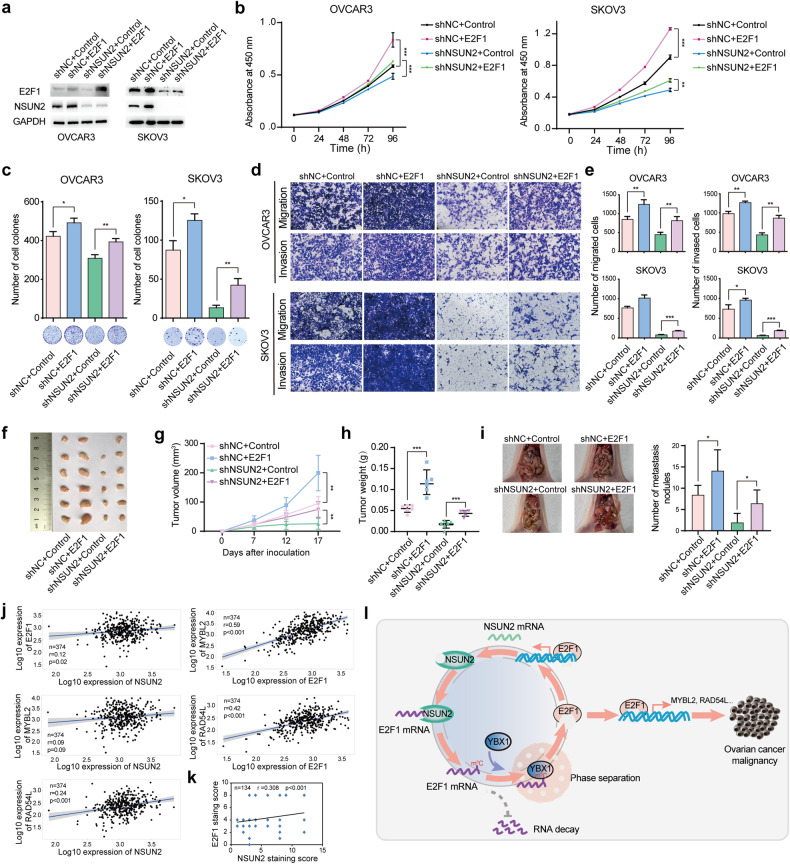
Ectopic expression of E2F1 ameliorates the suppressive effect of NSUN2 deficiency on ovarian cancer. **a** Western blot showing E2F1 expression in NSUN2-deficient ovarian cancer cells with re-expression of E2F1. **b** CCK-8 assay results showing the growth of NSUN2-deficient ovarian cancer cells with forced E2F1 expression. **c** E2F1 re-expression in NSUN2-deficient ovarian cancer cells promoted colony formation. **d**, **e** Effect of forced E2F1 expression on the migration and invasion of NSUN2-deficient ovarian cancer cells. **f**–**h** Tumor growth from ovarian cancer cells with or without NSUN2 overexpression followed by E2F1 overexpression was assessed by using nude mouse xenograft models. The volume and weight of the tumors formed were measured. **i** The metastasis of ovarian cancer cells with or without NSUN2 overexpression followed by E2F1 overexpression to the peritoneal cavity of mice was assessed. **j** Expression correlation analysis between NSUN2 and E2F1, NSUN2 and MYBL2, and NSUN2 and RAD54L, as well as between E2F1 and MYBL2 and E2F1 and RAD54L. **k** Spearman’s rank correlation analysis between NSUN2 and E2F1 protein expression in ovarian cancer patients (*n* = 134), as determined via tissue microarray analysis. **l** Proposed model of the NSUN2-E2F1 regulatory network in ovarian cancer. *p < 0.05, **p < 0.01, ***p < 0.001.

To further validate the implication of E2F1 in NSUN2-mediated ovarian cancer progression, we performed rescue assays in NSUN2-deficient ovarian cancer cells. Knockdown of NSUN2 suppressed E2F1 expression, but ectopic expression of E2F1 restored E2F1 expression, as shown by western blotting (Fig. 7a). Subsequently, CCK-8 and colony formation assays were conducted to evaluate cell growth, and transwell assays were performed to assess cell migration and invasion. We found that forced expression of E2F1, through which E2F1 expression was completely restored, restored the growth, migration, and invasion of NSUN2-deficient OVCAR3 cells (Fig. 7b–e). E2F1 expression was also partially restored in NSUN2-deficient SKOV3 cells (Fig. 7a). Consistent with the above results, the suppression of cell growth, migration, and invasion induced by NSUN2 deficiency was significantly ameliorated in SKOV3 cells, albeit to a lesser extent than in OVCAR3 cells (Fig. 7b–e). In vivo assays also showed that forced expression of E2F1 reversed the suppression of tumor growth and metastasis induced by NSUN2 deficiency (Fig. 7f–i). Furthermore, we analyzed the correlation between the expression of NSUN2 and E2F1 in ovarian cancer. NSUN2 expression was positively correlated with E2F1 expression, and E2F1 expression was also positively correlated with the expression of MYBL2 and RAD54L (Fig. 7j). Despite the negligible correlation between NSUN2 and MYBL2 expression, NSUN2 expression was positively associated with RAD54L expression (Fig. 7j). YBX1 expression was also positively associated with the expression of MYBL2 and RAD54L, albeit with a marginal correlation between YBX1 and E2F1 expression (Supplementary Fig. 11a). We also analyzed the correlation between NSUN2 protein and E2F1 protein expression and found a positive correlation (Fig. 7k). Additionally, survival analysis revealed that ovarian cancer patients with higher E2F1 expression tended to have shorter overall survival and progression-free survival times (Supplementary Fig. 11b). Despite the lack of a significant correlation between RAD54L expression and overall survival, higher MYBL2 and RAD54L expression tended to predict a worse prognosis in patients with ovarian cancer (Supplementary Fig. 11c, d). Taken together, these data support the hypothesis that NSUN2 promotes the m^5^C modification and hence the expression of E2F1 mRNA and that E2F1 reciprocally activates NSUN2 transcription, which leads to the formation of a positive feedback regulatory loop that drives the malignant progression of ovarian cancer (Fig. 7l).

## Discussion

In this study, we found that the methyltransferase *NSUN2* gene was amplified and upregulated in ovarian cancer and that NSUN2-induced RNA methylation facilitated the malignancy of ovarian cancer. RNA modifications have been demonstrated to be extensively involved in cancer occurrence and development^6–8^. In particular, the role of m^6^A, the most abundant RNA modification, has been widely investigated in multiple cancers^7^. In ovarian cancer, the m^6^A readers YTHDF1 and YTHDF2 specifically recognize RNA transcripts with m^6^A modification and affect their translation and decay, respectively^5,50^. m^6^A methyltransferases and demethylases are also involved in ovarian cancer progression through m^6^A-dependent or m^6^A-independent regulation^11,14^. A previous study revealed that pseudouridine synthase 7 (PUS7), which catalyzes RNA pseudouridine modification, might be a potential biomarker for ovarian cancer, but the underlying mechanisms were not explored^51^. Our previous analysis of m^5^C modification genes in ovarian cancer showed that a signature constructed based on m^5^C modification genes was able to predict poor prognosis in patients with ovarian cancer^52^. Consistently, our results revealed that several m^5^C modification genes were dysregulated in ovarian cancer, among which NSUN2 promoted ovarian cancer progression through regulating cell proliferation and metastasis, as we demonstrated. Importantly, we systematically mapped the m^5^C sites in mRNAs in ovarian cancer cells and found that the mRNAs of multiple transcription factors underwent m^5^C modification. m^5^C modification of E2F1 mRNA directs its regulation of target gene expression, which is implicated in the NSUN2-mediated tumor-promoting effect on ovarian cancer.

Although m^5^C modification was initially found on mRNA, more research has focused on the functions of m^5^C on tRNA or rRNA^17,53–55^. Recently, with advances in high-throughput techniques, m^5^C modifications on a large number of mRNAs and noncoding RNAs have been identified^16^. By RNA bisulfite sequencing, m^5^C modifications were mapped globally to total and nuclear poly(A) RNAs in embryonic stem cells (ESCs) and the murine brain^56^. A total of 7541 and 2075 candidate m^5^C sites were identified in ESCs and the brain, respectively, and substantial differences in the m^5^C prevalence and distribution among these sites were revealed^56^. Via meRIP with an antibody specific for m^5^C followed by high-throughput sequencing, 18,324 m^5^C peaks in 10,200 mRNAs and 21,960 m^5^C peaks in 19,935 lncRNAs were found in human hepatocellular carcinoma tissues^28^. We identified 16,536 m^5^C peaks in 6912 RNAs in control ovarian cancer cells compared with the corresponding NSUN2-knockdown cells. These m^5^C peaks were enriched in GC-enriched sequences, in agreement with findings in HeLa cells^21^. Moreover, NSUN2 knockdown caused a decrease in the global RNA m^5^C level. A majority of the RNAs with m^5^C peaks were protein-coding RNAs, although the m^5^C level of a considerable number of RNAs was increased in NSUN2-knockdown cells. In addition to NSUN2, NSUN6 and TRDMT1 have also been demonstrated to mediate m^5^C deposition on mRNA^19,20^. Knockdown of TRDMT1 in HEK293 cells resulted in 6772 differential m^5^C sites in mRNA, but the detailed changes in these m^5^C modifications were not shown^20^. However, NSUN6 primarily targets the 3′-UTR at the consensus sequence CTCCA and catalyzes the m^5^C modification of fewer mRNAs with sequence and structure specificity^19^.

NSUN2-mediated m^5^C modification can affect nuclear export, mRNA stability and mRNA translation through a process dependent on m^5^C reader proteins. As an adapter in the TREX-1 RNA export complex, ALYREF functions as a specific mRNA m^5^C-binding protein that modulates the export of mRNAs via m^5^C modification^21^. However, ALYREF binds to m^5^C-modified PKM2 mRNA and increases its stability in bladder cancer. YBX1 is also involved in maintaining the stability of m^5^C-modified mRNAs during bladder cancer development^22^. In *Drosophila*, the YBX1 homolog YPS preferentially stabilizes m^5^C-modified RNAs to promote ovarian germline stem cell development^57^. Lin28B and G3BP1 were also found to recognize m^5^C-modified RNAs and regulate their decay^23,26^. Through multiomics analysis, we found that NSUN2 positively regulated the m^5^C modification and expression of mRNAs. NSUN2 specifically interacted with the 3′-UTR of E2F1 mRNA, promoted its m^5^C modification, and increased its half-life. YBX1 was demonstrated to be the m^5^C reader controlling the stability of E2F1. Consistent with these findings, m^5^C modification within the 3′-UTR of mRNAs was previously demonstrated to play an important role in RNA stability, although more m^5^C sites were found in the CDS and 5′-UTR^24,58,59^. Thus, the m^5^C distribution in distinct regions of mRNAs and its role in gene regulation require additional characterization.

E2F1 is an essential oncogenic transcription factor involved in the progression of various cancers^44^. We found that knockdown of E2F1 substantially suppressed the proliferation and metastasis of ovarian cancer cells, whereas re-expression of E2F1 in NSUN2-deficient cells mitigated the suppressive effect of NSUN2 knockdown on cell proliferation and metastasis. Notably, NSUN2 was downregulated upon E2F1 knockdown. E2F1 affects NSUN2 transcription by interacting with the *NSUN2* promoter, thus suggesting that NSUN2 and E2F1 form a positive feedback loop in ovarian cancer. *NSUN2* is amplified, and its amplification is correlated with its increased RNA expression, which might be the trigger for the formation of the positive loop in ovarian cancer cells compared to normal ovarian epithelial cells. The NSUN2 amplification frequency (approximately 7%) is lower than the frequency of NSUN2 RNA upregulation (15%) in ovarian cancer, according to TCGA ovarian cancer datasets (Supplementary Fig. 1a–f). E2F1 upregulates NSUN2 expression by activating its transcription, suggesting that genetic regulation and transcriptional regulation synergistically contribute to NSUN2 upregulation in ovarian cancer. We also found that E2F1 modulated the transcription of MYBL2 and RAD54L through binding to their promoters. We also confirmed that similar to the effect of E2F1 on MYBL2 and RAD54L, knockdown of NSUN2 or YBX1 decreased the expression of MYBL2 and RAD54L, suggesting that MYBL2 and RAD54L are the downstream targets of NSUN2 and E2F1. Moreover, we analyzed the correlations among the expression of these genes. MYBL2 and RAD54L have been demonstrated to be downstream targets of E2F1, which has been demonstrated to be involved in tumor growth and metastasis. Intriguingly, YBX1 knockdown had little effect on NSUN2 expression, suggesting that other factors in addition to YBX1-mediated regulation of E2F1, MYBL2 and RAD54L expression might be involved in YBX1 regulation. The links between NSUN2-mediated RNA modification and transcriptional regulation by E2F1 prove the complexity of regulatory network underlying the malignancy of ovarian cancer.

In conclusion, our study revealed that the RNA methyltransferase NSUN2 is highly expressed in ovarian cancer and predicts a poor prognosis. NSUN2 promotes E2F1 expression in an m^5^C-dependent manner that is also dependent on YBX1 phase separation, and E2F1 can regulate NSUN2 transcription, thus forming the NSUN2-E2F1-NSUN2 feedback loop and providing a promising target for ovarian cancer therapy.

### Supplementary information

SUPPLEMENTAL MATERIAL

## Data Availability

All the datasets generated in this work have been deposited in the Sequence Read Archive (SRA) under accession numbers PRJNA828623, PRJNA830874, PRJNA831287 and PRJNA831153, and other data are available from the corresponding author upon reasonable request.
