# Supplementary material for: RNA m5C modification upregulates E2F1 expression in a manner dependent on YBX1 phase separation and promotes tumor progression in ovarian cancer
Source: Exp Mol Med. 2024 Mar 1;56(3):600–15. doi: 10.1038/s12276-024-01184-4 (PMC10984993; doi:10.1038/s12276-024-01184-4)
Supplement: Supplementary file 1 — SUPPLEMENTAL MATERIAL [file 12276_2024_1184_MOESM1_ESM.pdf]

Supplementary Information

RNA m5C modification upregulates E2F1 expression in a manner dependent on YBX1 phase separation and promotes tumor progression in ovarian cancer

Xiaoyi Liu, Qinglv Wei, Chenyue Yang, Hongyan Zhao, Jie Xu, Youchaou Mobet, Qingya Luo, Dan Yang, Xinzhao Zuo, Ningxuan Chen, Yu Yang, Li Li, Wei Wang, Jianhua Yu, Jing Xu, Tao Liu and Ping Yi

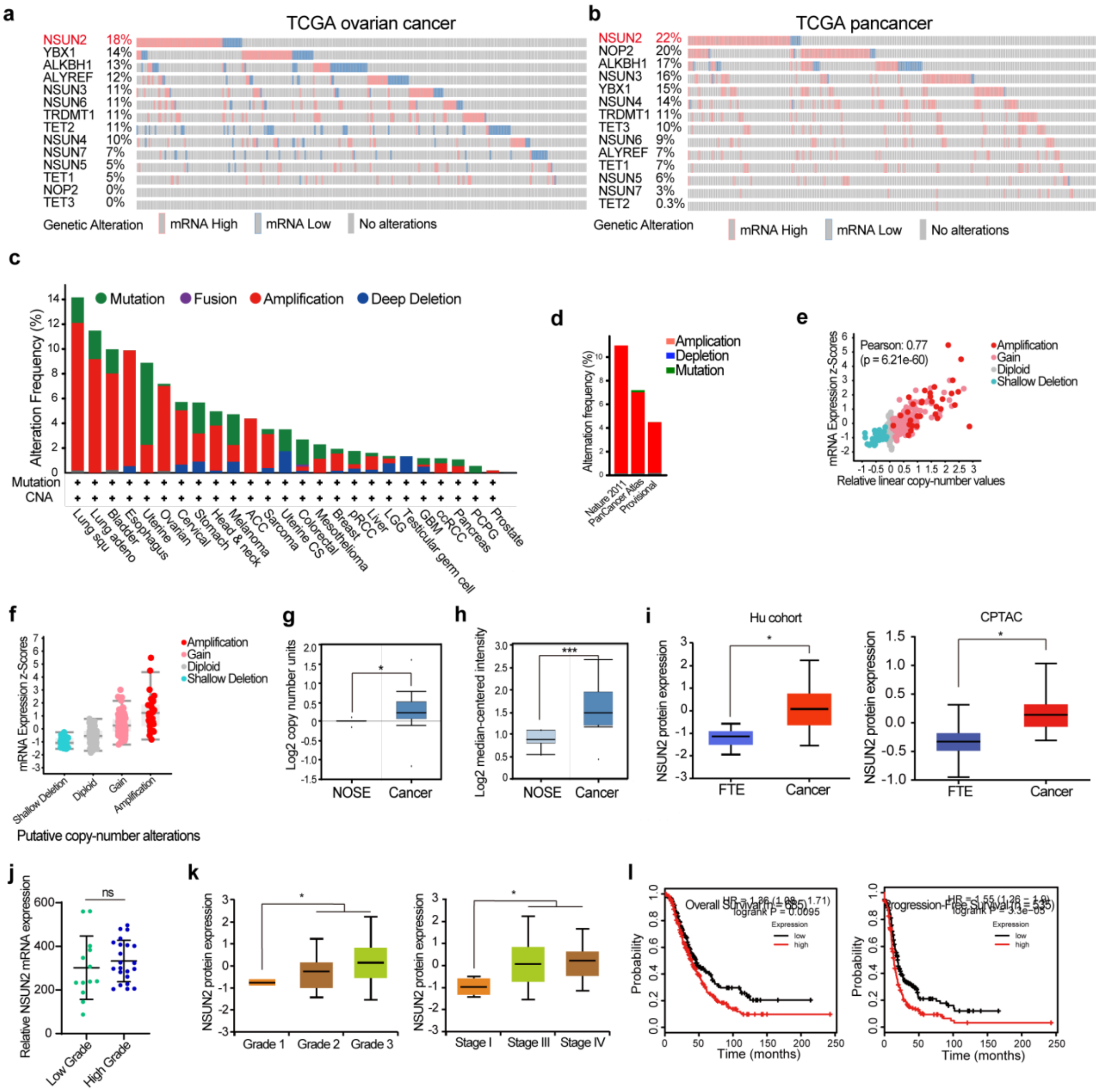

Supplementary Fig. 1 NSUN2 is highly expressed in ovarian cancer.

(a) RNA expression of m^{5}C regulators in the TCGA ovarian cancer dataset.

(b) RNA expression of m^{5}C regulators in the TCGA pan-cancer dataset.

(c) Genetic alternations of NSUN2 in cancers according to the TCGA pan-cancer dataset.

(d) Genetic alterations of NSUN2 in ovarian cancer datasets according to the TCGA database.

(e and f) Correlation analysis between the copy-number alterations of NSUN2 and its RNA expression.

(g) Copy number of NSUN2 in ovarian cancer according to the Oncomine database.

(h) RNA expression of NSUN2 in ovarian cancer according to the Oncomine database.

(i) NSUN2 protein expression in ovarian cancer compared with FTE in the Hu cohort and CPTAC database.

(j) RNA expression of NSUN2 in different grades of ovarian cancer according to GSE27651.

(k) Protein expression of NSUN2 in different grades or stages of ovarian cancer according to the CPTAC database.

(l) Kaplan-Meier analysis of the associations between NSUN2 expression and overall survival as well as progression-free survival of patients with ovarian cancer.

*$p < 0.05$, ns, not significant.

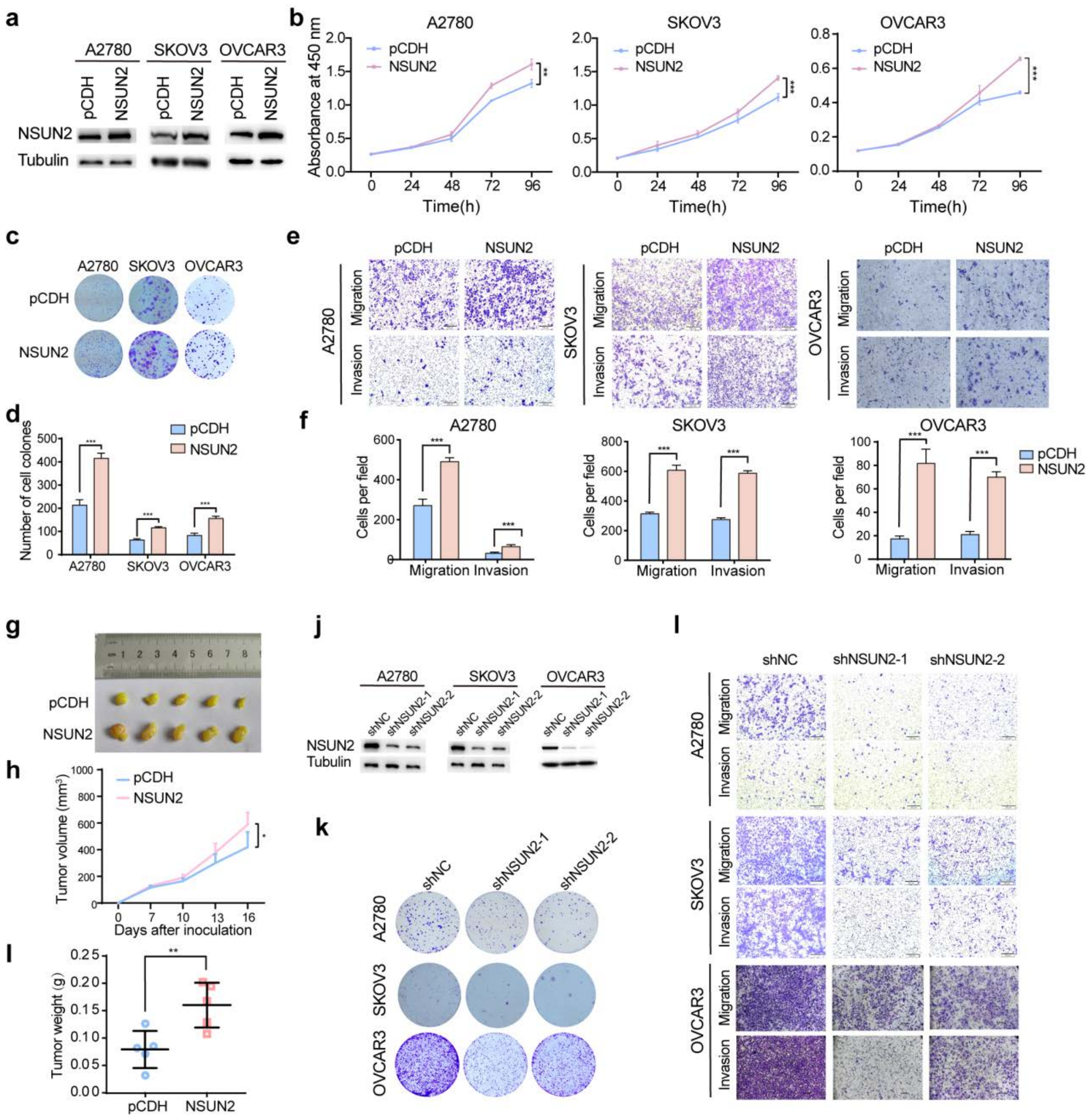

Supplementary Fig. 2 NSUN2 promotes ovarian cancer progression.

(a) Western blot was performed to confirm the overexpression of NSUN2 in ovarian cancer cells.

(b) CCK8 assays detecting the growth of ovarian cancer cells following forced expression of NSUN2.

(c and d) Colony-formation assays displaying the effect of NSUN2 overexpression on ovarian cancer cells' growth.

(e and f) Transwell analysis of migration and invasion of ovarian cancer cells upon NSUN2 overexpression.

(g-i) Tumorigenesis of ovarian cancer cells with NSUN2 overexpression assessed by

using the nude mouse xenograft models. The volume and weight of tumors formed were measured.

(j) NSUN2 protein levels in NSUN2-deficient A2780, SKOV3, and OVCAR3 ovarian cancer cells were assessed by western blot.

(k) Colony-formation assays displaying the effect of NSUN2 knockdown on ovarian cancer cells' growth.

(l) Transwell analysis of migration and invasion of ovarian cancer cells with or without NSUN2 expression.

**p< 0.01, ***p< 0.001.

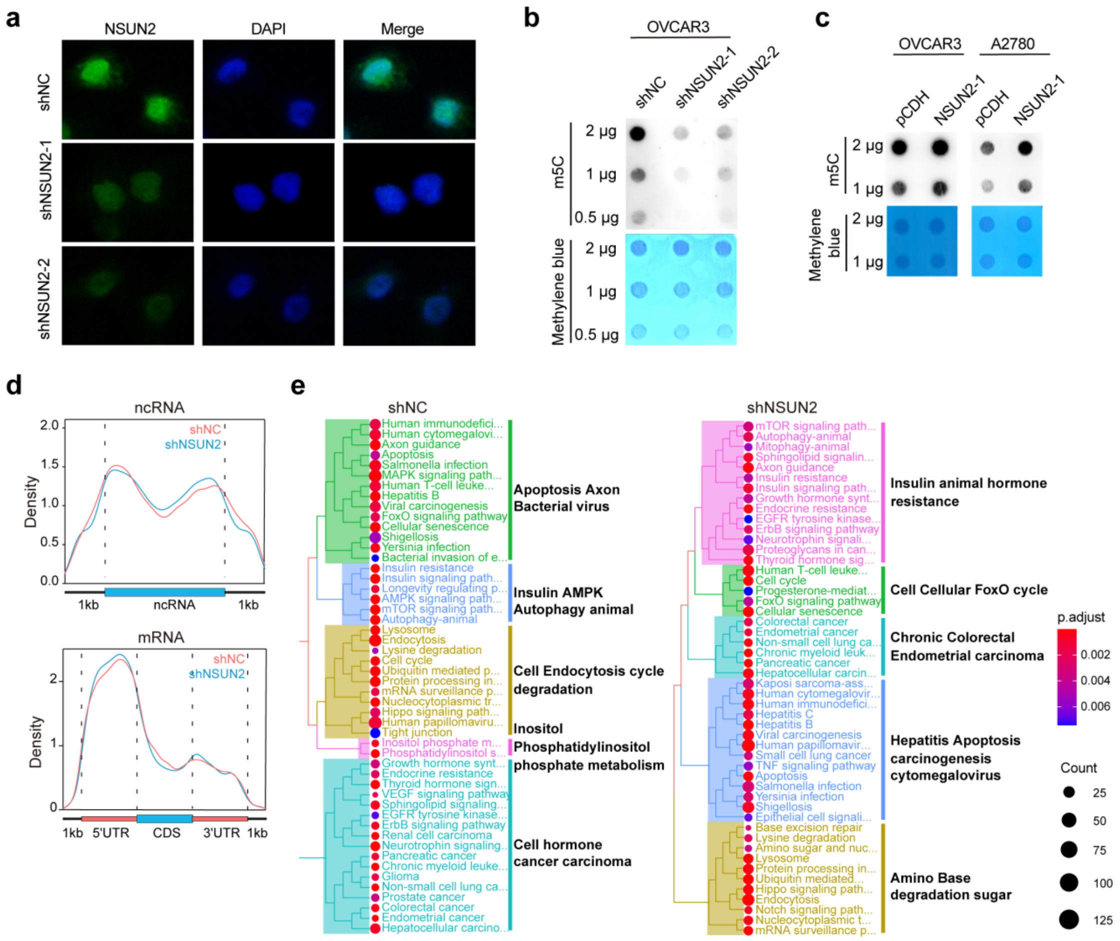

Supplementary Fig. 3 Related to Figure 2.

(a) Immunofluorescence detection of NSUN2 expression in ovarian cancer cells following NSUN2 knockdown.

(b) Dot blot detecting m^{5}C modification of global mRNA in OVCAR3 cells upon NSUN2 knockdown.

(c) Dot blot detecting m^{5}C modification of global mRNA in OVCAR3 and A2780 cells upon NSUN2 overexpression.

(d) Metagene profiling of m^{5}C sites within noncoding RNA and mRNA in control and NSUN2-knockdown cells, respectively.

(e) Function annotations of hypermethylated m^{5}C peaks in ovarian cancer cells with or without NSUN2 by GO analysis.

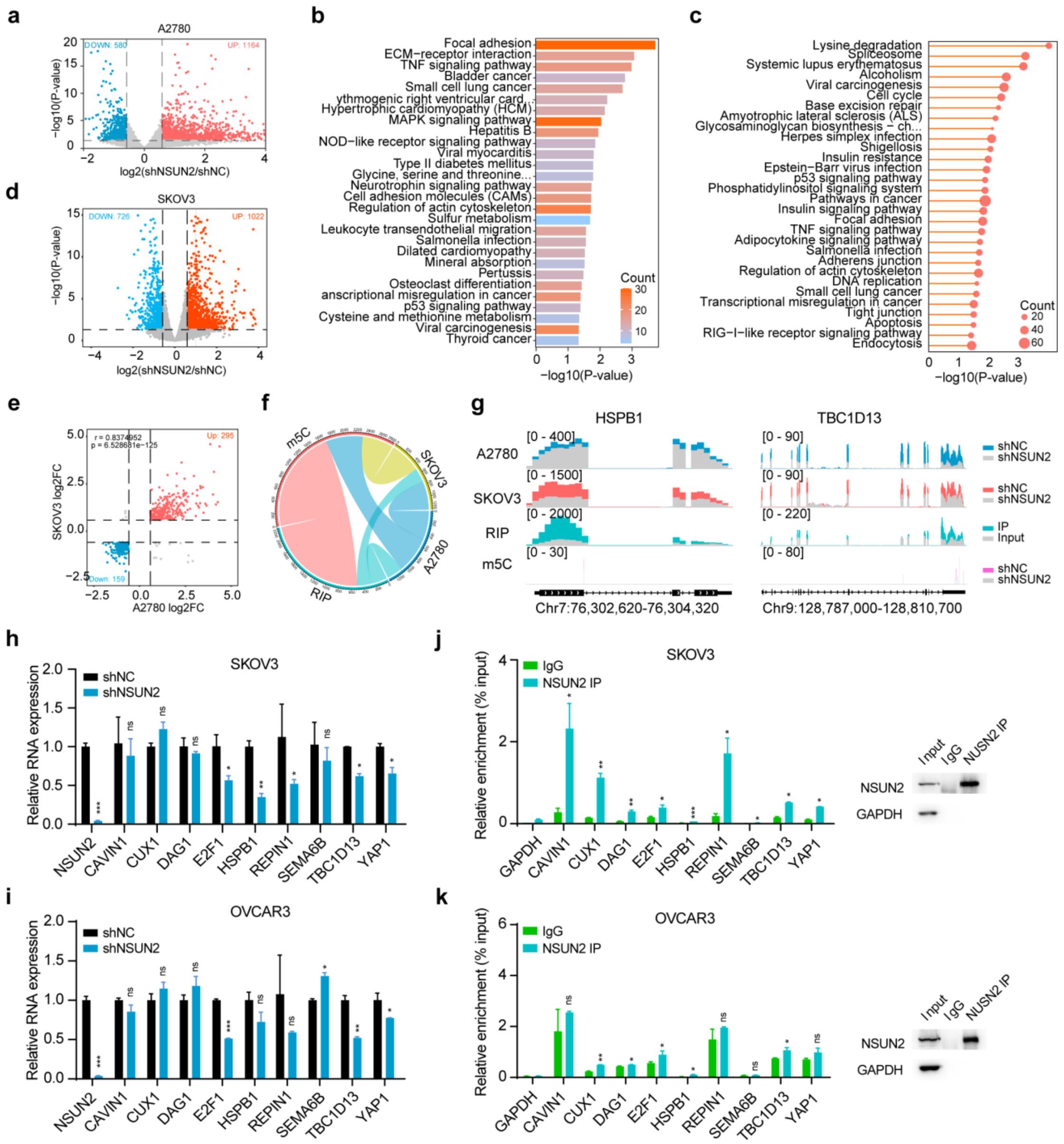

Supplementary Fig. 4 Related to Figure 3.

(a) Volcano plot showing DEGs in A2780 cells upon NSUN2 knockdown.

(b) GO functional enrichment analysis of DEGs shown in (a).

(c) GO functional enrichment analysis of NSUN2-bound transcripts.

(d) Volcano plot showing DEGs in SKOV3 cells upon NSUN2 knockdown.

(e) Correlations of DEGs in A2780 and SKOV3 cells upon NSUN2 knockdown.

(f) Circus plot displaying the overlapping of multi-omics data.

(g) Metaplot showing the distribution of m^{5}C sites and RNA abundance of HSPB1 and TBC1D13 in ovarian cancer cells upon NSUN2 knockdown as well as NSUN2 binding in ovarian cancer cells.

(h and i) RT-PCR detecting RNA levels upon NSUN2 knockdown in SKOV3 and OVCAR3 cells.

(j and k) RIP-PCR confirming the interaction between NSUN2 and targeted transcripts in SKOV3 and OVCAR3. Proteins immunoprecipitated were detected by western blot.

*$p < 0.05$, **$p < 0.01$, ***$p < 0.001$, ns, not significant.

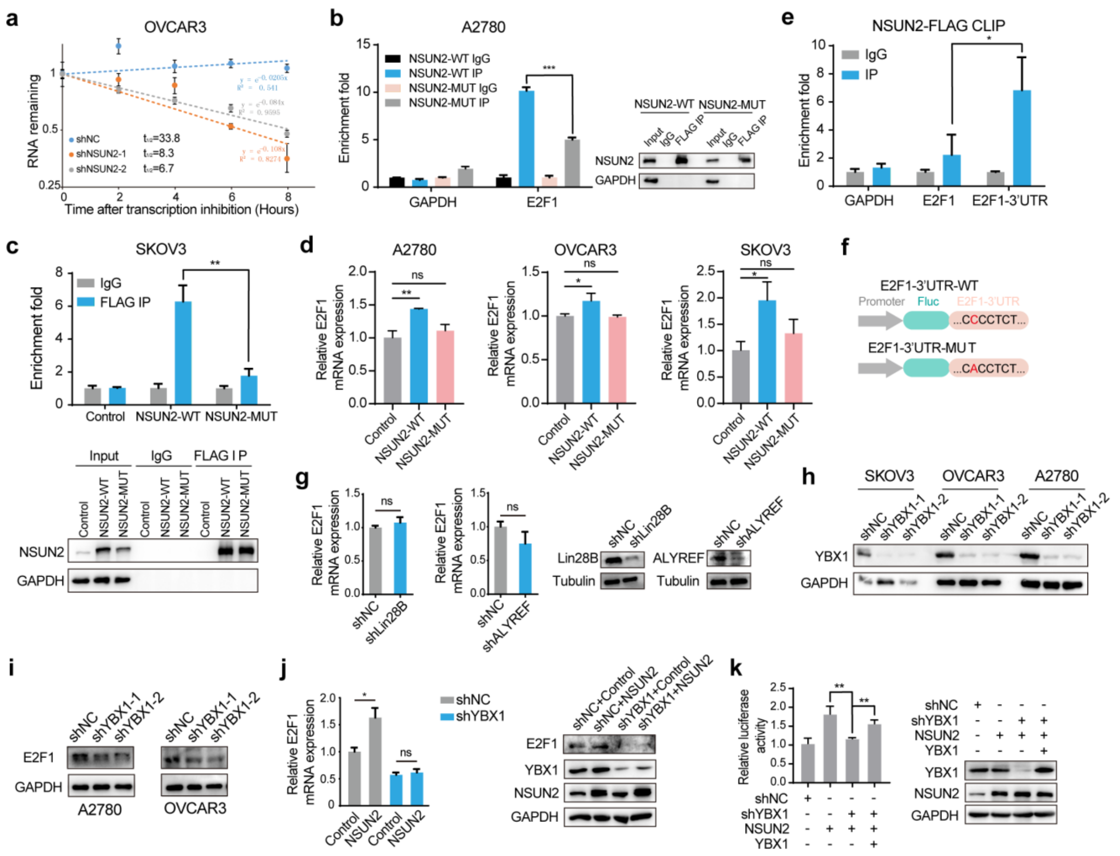

Supplementary Fig. 5 Related to Figure 4.

(a) The effect of NSUN2 knockdown on the half-life of E2F1 mRNA in OVCAR3 cells.

(b and c) RIP-PCR confirmed the interactions of NSUN2 or NSUN2 mutant with E2F1 mRNA in A2780 (b) and OVCAR3 cells (c). Protein immunoprecipitated by using the antibody to FLAG were assessed by western blot.

(d) RNA expression of E2F1 was detected in ovarian cancer cells upon wild-type or mutated NSUN2 overexpression.

(e) eCLIP assay detecting the interaction between NSUN2 and 3’-UTR of E2F1 mRNA.

(f) Schema of the reporter carrying E2F1 3’-UTR with either wild-type or m^{5}C site deletion.

(g) E2F1 expression was measured upon ALYREF or Lin28B knockdown in OVCAR3 ovarian cancer cells.

(h) YBX1 protein levels in YBX1-deficient A2780, SKOV3, and OVCAR3 ovarian cancer cells were assessed by western blot.

(i) E2F1 protein levels in YBX1-deficient A2780, SKOV3, and OVCAR3 ovarian cancer cells were assessed by western blot.

(j) E2F1 expression was assessed in ovarian cancer cell upon YBX1 kncokdown followed by NSUN2 overexpression.

(k) Relative luciferase activity of the reporter carrying E2F1 3'-UTR in cells with NSUN2 overexpression and YBX1 knockdown as well as YBX1 re-expression was detected and normalized to renilla luciferase activity. Western blot assays confirmed the expression of NSUN2 and YBX1 in the indicated cells.

*$p < 0.05$, **$p < 0.01$, ***$p < 0.001$, ns, not significant.

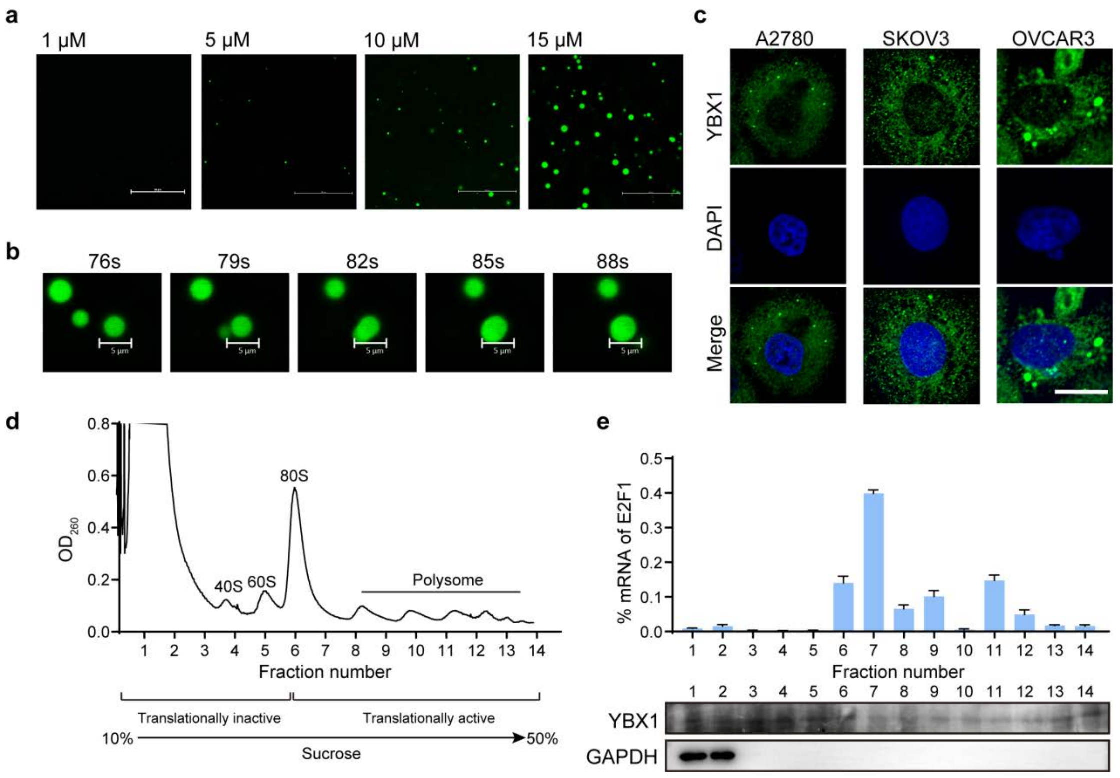

Supplementary Fig. 6 Related to Figure 5.

(a) Phase separation of YBX1 at different concentrations. Scale bar, 50 μm.

(b) Time-lapse images of YBX1 droplets showing a droplet fusion. Scale bar, 5 μm.

(c) Immunofluorescence imaging of YBX1 in ovarian cancer cells shows cytoplasmic YBX1 puncta. Scale bar, 10 μm.

(d and e) Polysome profiling in A2780 ovarian cancer cells (d), and expression of E2F1 mRNA and YBX1 protein expression in each fraction were detected by RT-qPCR and western blot, respectively (e).

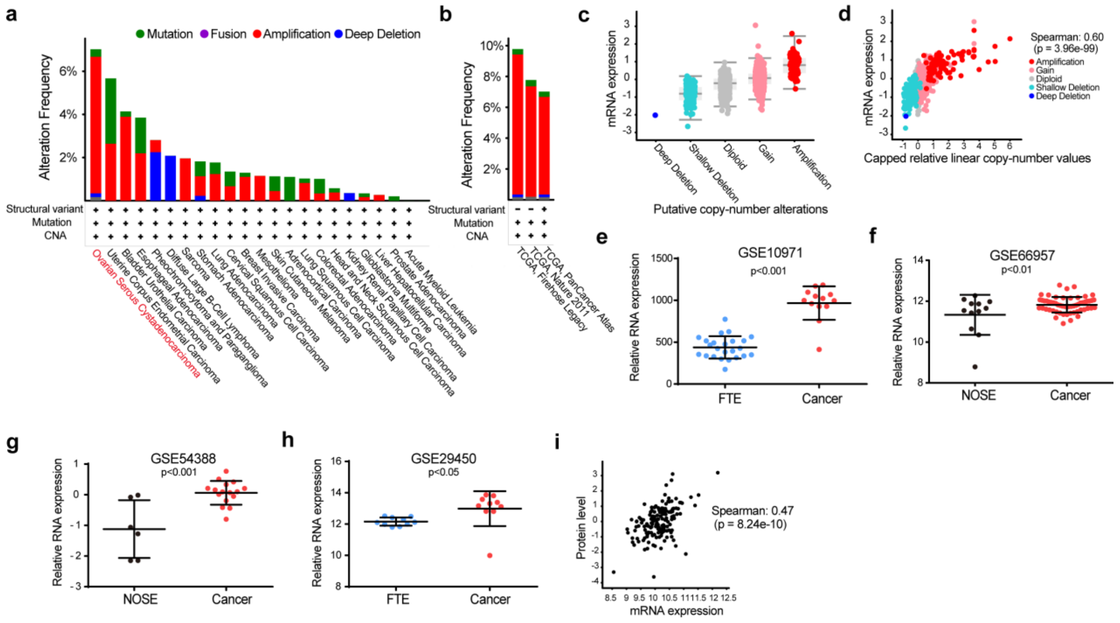

Supplementary Fig. 7 YBX1 is upregulated in ovarian cancer.

(a) Genetic alterations of YBX1 in cancers according to the TCGA pan-cancer dataset.

(b) Genetic alterations of YBX1 in the TCGA ovarian cancer datasets.

(c and d) Correlation analysis between the copy number of YBX1 and its RNA expression.

(e-h) RNA expression of YBX1 in ovarian cancer according to GEO datasets.

(i) Correlation analysis between the RNA level of YBX1 and its protein expression.

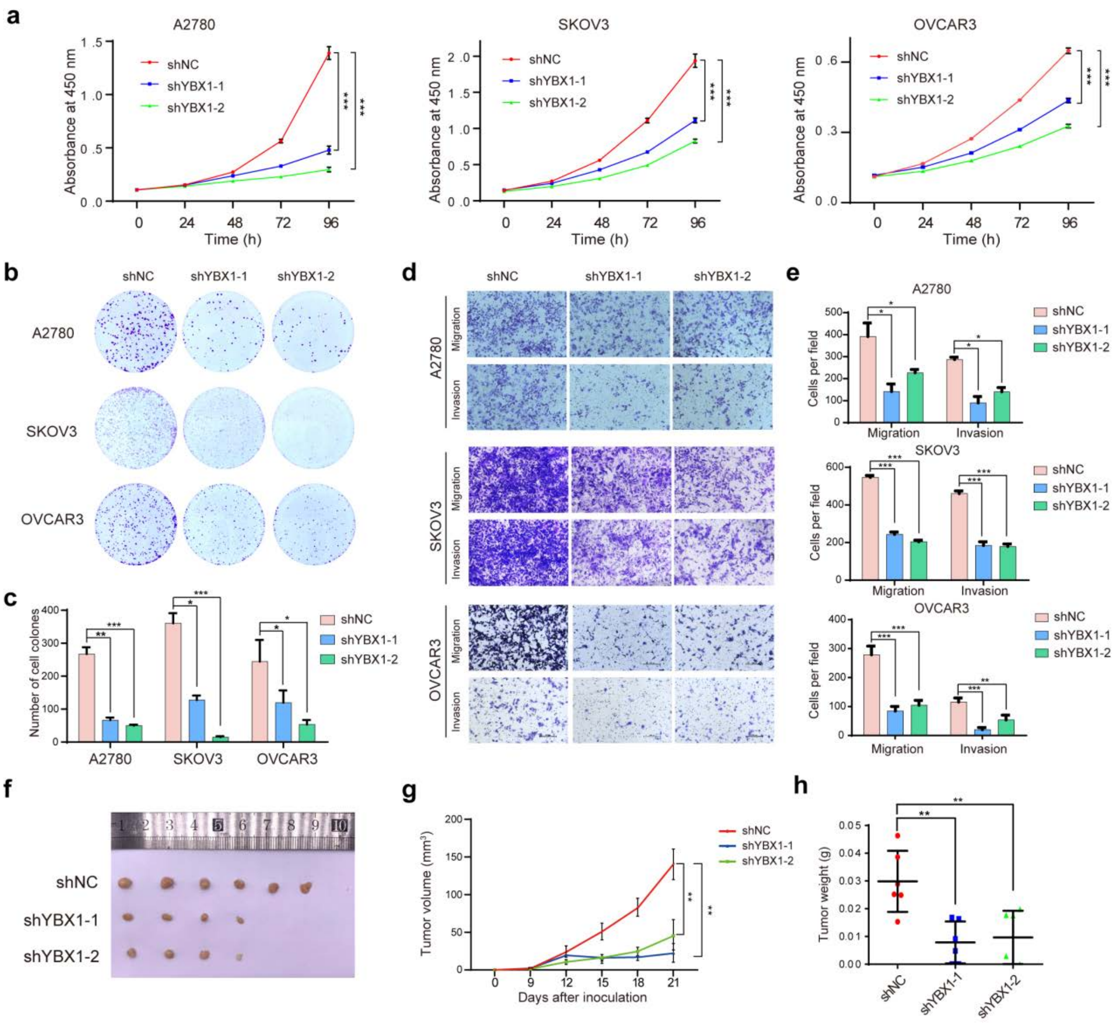

Supplementary Fig. 8 YBX1 promotes ovarian cancer progression.

(a) CCK8 assays detecting the growth of ovarian cancer cells upon YBX1 knockdown.

(b and c) Colony-formation assays displaying the effect of YBX1 knockdown on ovarian cancer cells' growth.

(d and e) Transwell analysis of migration and invasion of ovarian cancer cells with or without YBX1 expression.

(f-h) Tumorigenesis of A2780 ovarian cancer cells with or without YBX1 expression was assessed by using the nude mouse xenograft models.

**p*< 0.05, ***p*< 0.01, ****p*< 0.001.

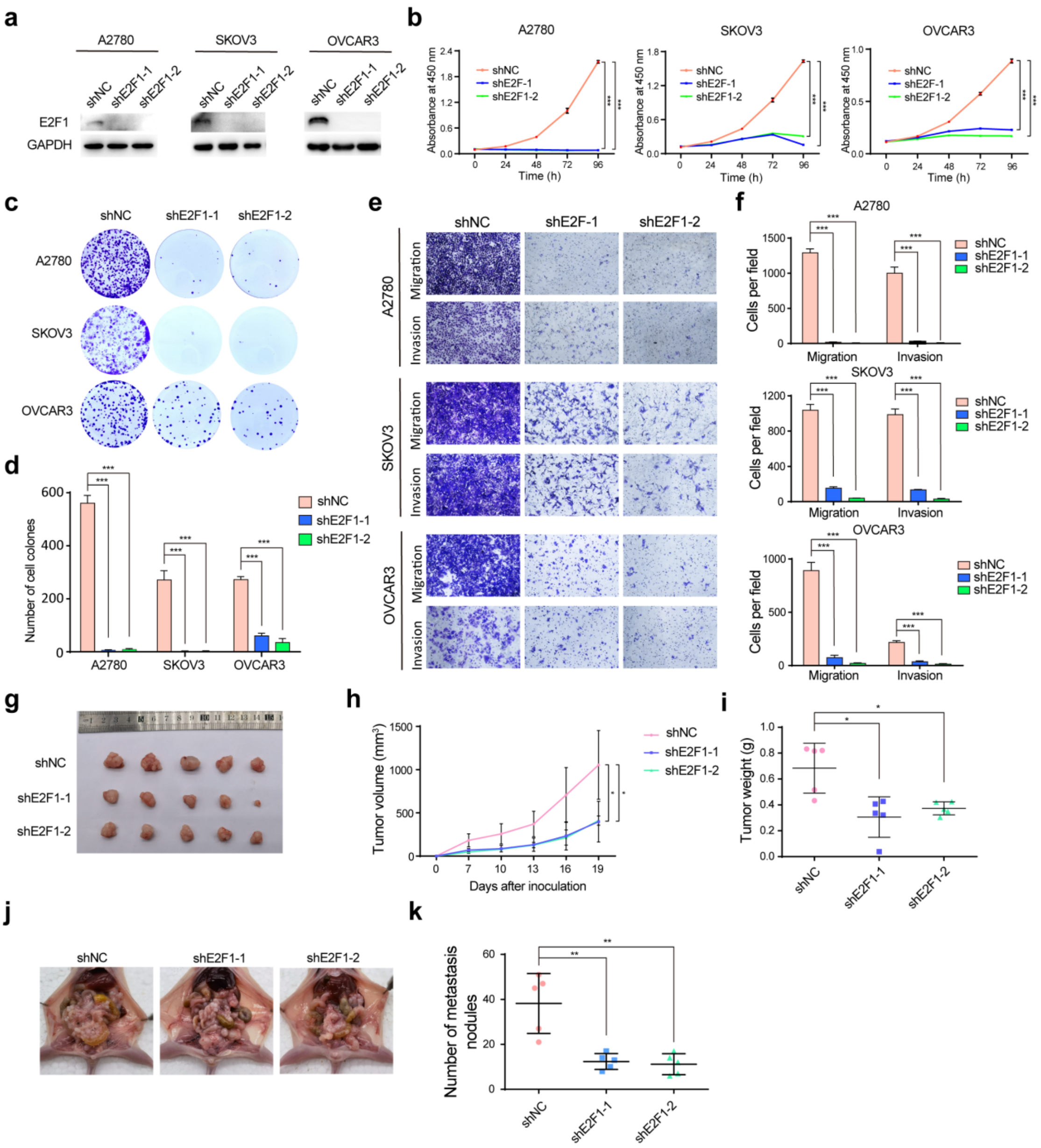

Supplementary Fig. 9 E2F1 promotes the malignant progression of ovarian cancer.

(a) Western blot detecting E2F1 protein expression in ovarian cancer cells with E2F1 depletion.

(b) Loss of E2F1 prevented the growth of ovarian cancer cells by CCK-8 assays.

(c and d) E2F1 knockdown retarded the colony-formation of ovarian cancer cells.

(e and f) E2F1 knockdown inhibited the migration and invasion of ovarian cancer cells.

(g-i) E2F1 depletion impeded the tumorigenesis of ovarian cancer cells by nude mouse xenograft models.

(j and k) E2F1 depletion suppressed the metastasis of ovarian cancer cells in abdominal cavity of mice.

$^{*}p < 0.05$, $^{**}p < 0.01$, $^{***}p < 0.001$.

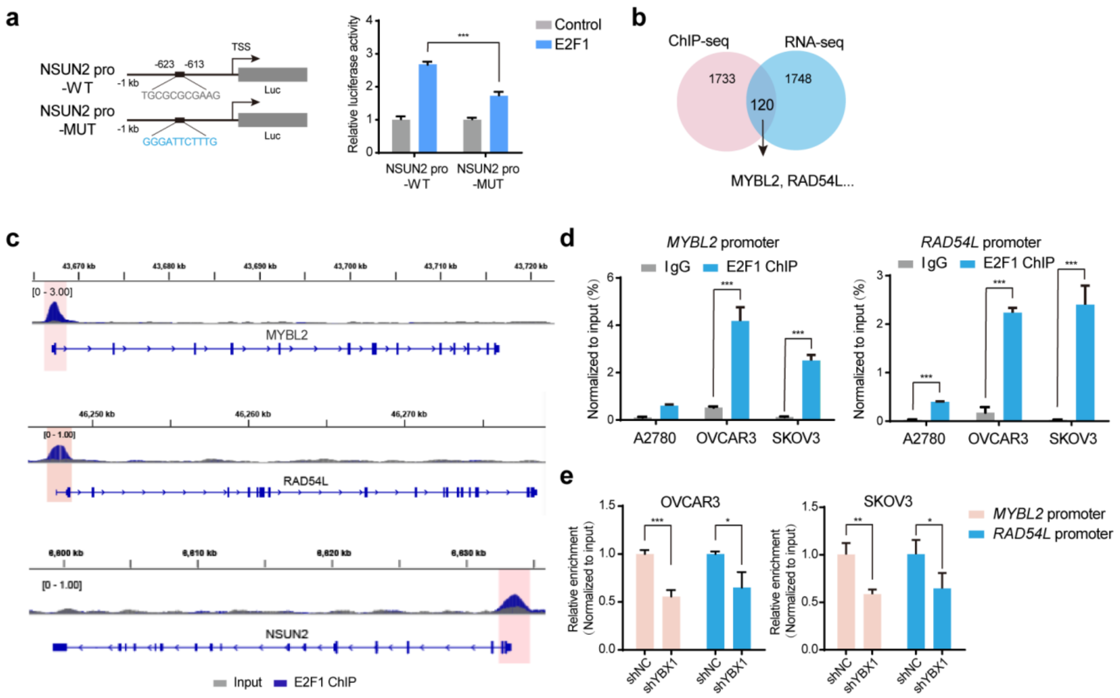

Supplementary Fig. 10 Related to Figure 6.

(a) Schemic diagram of the reporter carrying NSUN2 promoter with E2F1 binding site wild-type or mutant. Relative luciferase activity of the reporter carrying NSUN2 promoter with E2F1 binding site wild-type or mutant in cells upon E2F1 overexpression was detected and normalized to renilla luciferase activity.

(b) Venn diagram showing the overlapping genes from E2F1 ChIP-seq and RNA-seq upon NSUN2 knockdown.

(c) IGV diagram showing the binding sites of E2F1 at the promoters of *MYBL2*, *RAD54L* and *NSUN2*.

(d) ChIP assays of E2F1 detecting that E2F1 bound to the promoters of *MYBL2* and *RAD54L* in ovarian cancer cells.

(e) Effect of YBX1 knockdown on the E2F1 binding at the promoters of *MYBL2* and *RAD54L* in ovarian cancer cells.

**p*< 0.05, ***p*< 0.01, ****p*< 0.001.

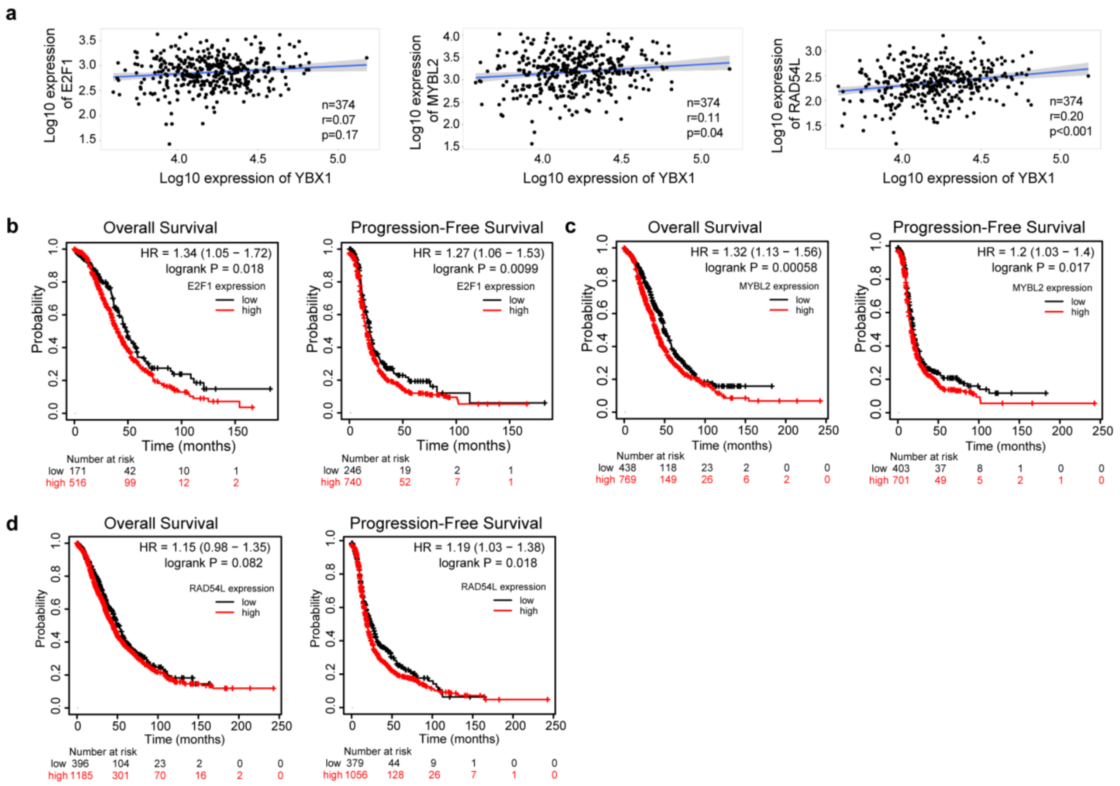

Supplementary Fig. 11 Related to Figure 7.

(a) Correlation analysis between YBX1 expression and E2F1, MYBL2 and RAD54L expression in ovarian cancer.

(b-d) Associations of E2F1, MYBL2 and RAD54L expression with overall survival and progression-free survival, respectively.

Supplementary Table 1. Sequences of primers and oligos used in this study

**Oligonucleotides**	**Sequences**
shNSUN2-1	GCGGCTTCATTATCTCAGGAT
shNSUN2-2	CCTGAAGATGATCCTTTATTT
shYBX1-1	CCAGTTCAAGGCAGTAAATAT
shYBX1-2	AGCAGACCGTAACCATTATAG
shE2F1-1	ACATCACCAACGTCCTTGAG
shE2F1-2	CGTGGACTCTTCGGAGAACTT
NSUN2-F	GCTACCCCGAGATCGTCAAG
NSUN2-R	TCAGGATACCTTTTGTAACCAGT
GAPDH-F	TGCACCACCAACTGCTTAGC
GAPDH-R	GGCATGGACTGTGGTCATGAG
E2F1-F	CATCCCAGGAGGTCACTTCTG
E2F1-R	GACAACAGCGGTTCTTGCTC
CAVIN1-F	GGGCCGTAGACCAGATCCA
CAVIN1-R	CTTGCTCACCGTATTGCTCGT
CUX1-F	GAAGAACCAAGCCGAAACCAT
CUX1-R	AGGCTCTGAACCTTATGCTCA
DAG1-F	TCGAGTGACCATTCCAACAGA
DAG1-R	GCACACCCTTATCAGTGTCAA
HSPB1-F	TGGACCCCACCCAAGTTTC
HSPB1-R	CGGCAGTCTCATCGGATTTT
REPIN1-F	GCCTTCTGTTGTGCCATCTGT
REPIN1-R	TCTCAGGCATCGTGCTTCTTCC
SEMA6B-F	AAGGTGCTGACGACCTCAAC
SEMA6B-R	CCTTCATCCGACACACGTTTATG
TBC1D13-F	GGAGGTTGTGCCCAGACATTT
TBC1D13-R	CCACTCTCTTACGAAGGGTTTCA
YAP1-F	TAGCCCTGCGTAGCCAGTTA
YAP1-R	TCATGCTTAGTCCACTGTCTGT
18SrRNA-F	CGATAACGAACGAGACTCTGGC
18SrRNA-R	CGGACATCTAAGGGCATCACA
E2F1-3'-UTR-F	ATGCTCACCTTGTCTCTGCAGC
E2F1-3'-UTR-R	TTCCCTTCCTGGCTTGCTCAG
